# An investigation into the effects and effectiveness of correlation network filtration methods with financial returns

**DOI:** 10.1371/journal.pone.0273830

**Published:** 2022-09-07

**Authors:** Tristan Millington

**Affiliations:** Usher Institute, University of Edinburgh, NINE Bioquarter, Edinburgh, United Kingdom; Universidad de Almeria, SPAIN

## Abstract

When studying financial markets, we often look at estimating a correlation matrix from asset returns. These tend to be noisy, with many more dimensions than samples, so often the resulting correlation matrix is filtered. Popular methods to do this include the minimum spanning tree, planar maximally filtered graph and the triangulated maximally filtered graph, which involve using the correlation network as the adjacency matrix of a graph and then using tools from graph theory. These assume the data fits some form of shape. We do not necessarily have a reason to believe that the data does fit into this shape, and there have been few empirical investigations comparing how the methods perform. In this paper we look at how the filtered networks are changed from the original networks using stock returns from the US, UK, German, Indian and Chinese markets, and at how these methods affect our ability to distinguish between datasets created from different correlation matrices using a graph embedding algorithm. We find that the relationship between the full and filtered networks depends on the data and the state of the market, and decreases as we increase the size of networks, and that the filtered networks do not provide an improvement in classification accuracy compared to the full networks.

## 1 Introduction

The study of correlation matrices estimated from financial data has been of interest to econo-physicists for many years. We do this as it allows us to quantify levels of risk in a portfolio. In general, we desire a low risk portfolio to consist of negatively correlated assets. This means that even if the value of one of the assets drops, the others will not. This involves calculating the correlation matrix from a set of stock returns. Most authors use Pearson correlation due to its interpretability, but other options are available, including partial correlation [1–3], rank correlation [4, 5] or mutual information [6]. Asset returns are generally not stationary [7], and so a small window of data is usually chosen where stationarity can be assumed [8]. However, the accurate estimation of a correlation matrix requires a relatively large window of data, as the correlation matrix will be ill-formed if there is more dimensions than samples. This therefore necessitates a trade off, with often the smaller window of data winning out. To overcome this issue, we can choose to estimate fewer edges by removing noisy ones. This has the added advantage of making the resulting correlation matrix easier to interpret.

**Fig 1:**
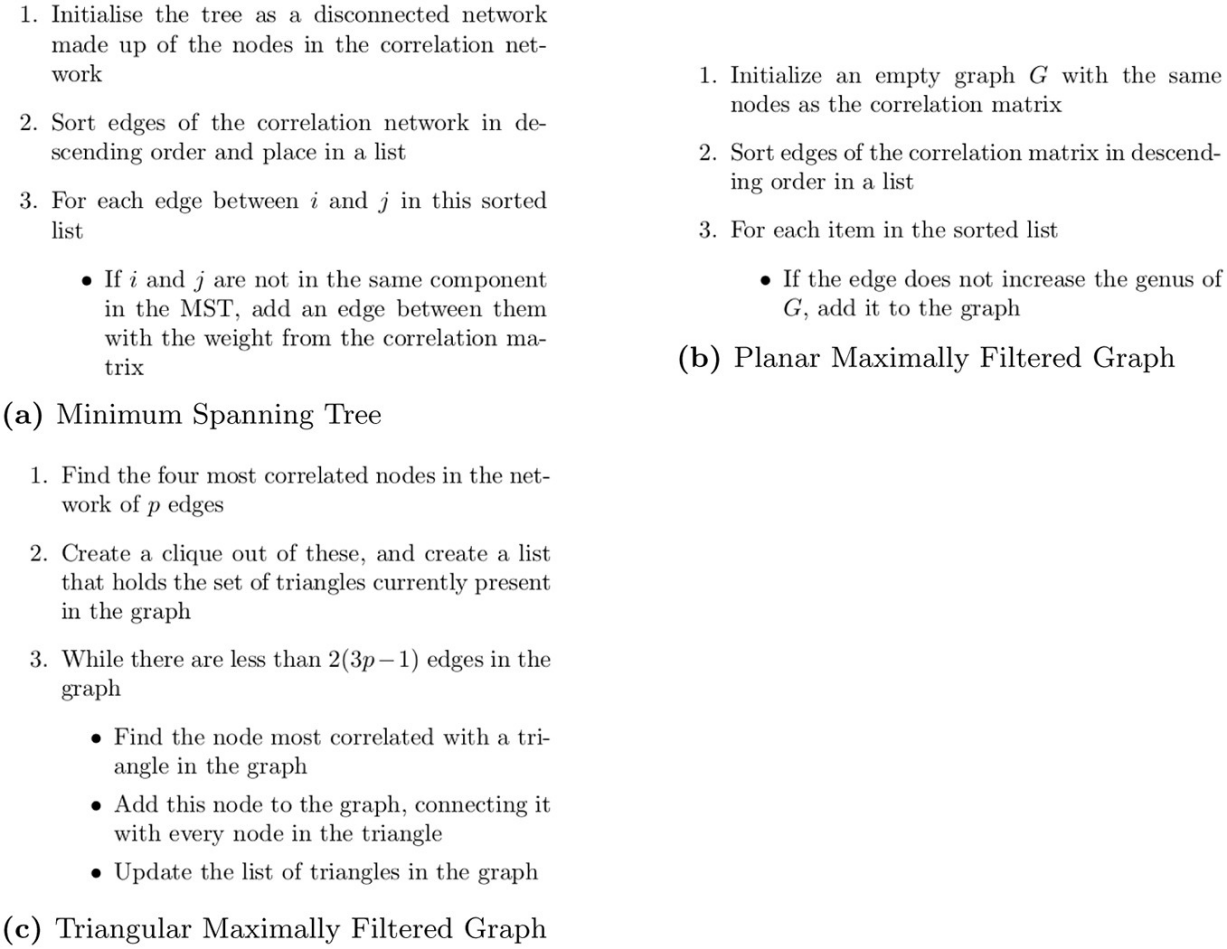
Pseudo code for the described topological filtration methods. (**a**) Minimum Spanning Tree, (**b**) Planar Maximally Filtered Graph, (**c**) Triangular Maximally Filtered Graph.

Deciding which edges are noisy is not a trivial task. Various methods have been proposed to solve this, such as sparsity [9, 10] or thresholding [11], but we are particularly interested in topological methods. Here we assume the dataset fits a certain shape, and keep the strongest edges that correspond to this shape. Once these edges have been removed, we can treat the correlation matrix as the adjacency matrix of a network, and use tools from graph theory to study it. This results in a financial network, with nodes in said network representing companies and edges representing relationships between them. The two most popular options for filtering a correlation matrix are the Minimum Spanning Tree (MST) [12, 13] and the Planar Maximally Filtered Graph (PMFG) [14]. Using the MST to filter a correlation matrix was originally proposed by Mantegna [12]. However, this method retains only a small number of the correlations present—from a matrix containing *p* assets only *p* − 1 edges are retained. Tumminello et al. [14] therefore proposed a method which retained more edges, the PMFG, which greedily selects the largest correlations while ensuring the resulting graph is planar (can be drawn on a 2d surface with no edges crossing). This results in 3*p* − 6 edges retained. However, the PMFG relies on a planarity check at each iteration, which is very computationally expensive for larger graphs. Therefore, Massara et al. [15] proposed the TMFG, which constructs a planar graph without the requirement for a planarity check. Instead, at each iteration the new node is added as a clique (a sub-graph where all nodes are fully connected to each other) into a triangular face. Pseudo code for each of these filtration methods can be found in Fig 1.

While much of the focus of the applications of these filtration methods has been on stock returns [13, 16], there have been applications on other types of data, including cryptocurrencies [17, 18], gene expression data [19, 20], fMRI data [21, 22] and on semantic networks [23].

While these methods do produce graphs with greater interpretability, there is not necessarily a great deal of evidence to believe that the underlying dataset can nicely fit into such structures [24]. Furthermore, these models also discard many correlations, which changes the structure of the resulting networks, sometimes in non-obvious ways [25]. There is also little evidence on whether these methods actually improve performance on certain tasks. We have only found one paper on this subject. In this paper, Tola et al. [26] find that using MST filtered correlation matrices does not improve portfolio optimization performance unless the volatilities are known beforehand.

Therefore in this paper we look at studying the effects of these filtration methods compared to just using the full correlation matrix. To do this we use a set of stock data from five different countries, the US, UK, Germany, India and China. Using this spread of countries also allows us to see if the effects can be seen in both developed and developing markets, as these are known to have different structures [27].

Firstly, we study how the filtered networks differ on a node and edge level from the full networks, and how this varies depending on the number of dimensions considered in the analysis. Secondly, we study how this difference changes with the market state. Thirdly, we look at using an objective measure to quantify how useful the methods are. In particular we focus on graph classification—if we create different underlying graphs, do the filtration methods cause an increase in accuracy compared to an unfiltered graph? Furthermore which filtration method gives the best accuracy? To the best of our knowledge, this approach to objectively evaluate the correlation filtration methods has not been undertaken before. We choose the task of graph classification for a few reasons. Firstly, we know the market tends to fit into certain states [28, 29], and therefore it should be possible to distinguish between these states using these networks. Secondly, graph classification is not specific to financial networks, and networks inferred from other sources can also be classified (as opposed to using the inferred correlation matrices for portfolio selection, for instance). Therefore this could extend to datasets from other fields, and help to show how these methods generalize.

## 2 Materials and methods

### 2.1 Correlation matrix creation

We create three ‘types’ of correlation matrix for our initial investigations into the effects of the filtration methods, using returns from the windows of minimum, maximum and median volatility. Each window is 504 days in size. The volatility is measured using the mean of the standard deviation of all the stock returns in the dataset during that window. From these windows, we take *p* dimensions at random to create a set of sub-datasets for each class. The values of *p* go from 10 to 200 for the US, 10 to 70 for the UK, 10 to 23 for DE, 10 to 45 for IN and 10 to 30 for CH inclusive, incrementing by 10 for the US, 5 for the UK, 5 for IN and 1 for DE and CH. We then calculate a set of sample correlation matrices from these sub-datasets, to obtain a dataset of correlation matrices belonging to the three classes, the minimum, maximum and median volatility windows.

We also look at doing some analysis on how the relationship between the filtered and full networks varies over time. To do this, we take windows of 504 days of data, and then slide along the dataset for 30 days at a time. We do not do any sampling for these datasets. From each window, we calculate the correlation matrix and then run it through each filtration method.

For the classification, we take a similar approach to the initial investigations. However for this we also vary the number of samples involved. For each value of *p* chosen, we generate 20 subdatasets by sampling from each full window without replacement for each value of *n* between 10 and 200, inclusive, incrementing by 10 each time. We calculate a correlation matrix for each subdataset, and use these as inputs into the classification procedure (detailed below).

### 2.2 Network analysis

To quantify the relationships between the full and filtered networks, we use two simple measures. The first is the Pearson correlation between the weighted degree centralities of each node. The weighted degree centrality of node *i* in a correlation network *C* is defined as the sum of the weights of the edges connected to said node:

$$\begin{array}{c} d_{i}=\sum_{i\neq j}C_{ij} \end{array}$$ (1)

Calculating this for every node in the network, we end up with a vector **d**. If we do this for the full network and then a filtered network, we can then calculate the Pearson correlation between these

$$\begin{array}{c} d_{\text{corr}}=\frac{\sum_{i=1}^{p}\left(d_{i}-\bar{d}\right)\left(d_{i}^{f}-\bar{d^{f}}\right)}{\sqrt{\sum_{i=1}^{p}{\left(d_{i}-\bar{d}\right)}^{2}{\left(d_{i}^{f}-\bar{d^{f}}\right)}^{2}}} \end{array}$$ (2)

where *d*_*i*_ is the degree of node *i* in the full correlation network, $d_{i}^{f}$ is the degree of node *i* in the filtered correlation network, $\bar{d}$ is the mean of the degree centralities in the network.

This is referred to as degree correlation or degree agreement in the rest of the paper. This simply measures if the networks agree on the same nodes being important.

The second measure is the Pearson correlation between the off diagonals of the adjacency matrices (as the diagonal of a correlation matrix always contains 1s). If we have two correlation matrices for the full (*C*) and filtered (*C*^*f*^) networks, we firstly turn the matrices into vectors in a row major style, discarding the diagonal, resulting in two vectors (**c**, **c**^**f**^). We can then calculate the Pearson correlation between these vectors as above. This is referred to as the edge correlation or edge agreement in the rest of the paper.

This measures the overlap of edges between the two networks, with edges with a larger weight being regarded as more important for this measure.

In general, if the filtration methods are discarding low valued edges which are noisy, while retaining those edges which are considered signal, we should expect that both of these measures should be relatively high.

When investigating how varying *p* affects the resulting networks, we will have a set of correlation matrices for each value of *p*. For each correlation matrix we then calculate the degree and edge agreements, and take the mean and standard deviation across the 20 runs for each *p*.

### 2.3 Graph classification

Graph classification is a fast moving field, with many new methods being proposed over the past few years. Various approaches have been taken, including kernel methods [30], embedding methods [31, 32], deep neural networks [33] or manual feature crafting [34]. In general most applications involving graph classification on real world graphs tend to involve manually extracting topological measures and then using these to classify graphs [35–37].

We use a very simple method, developed by de Lara et al. [32]. This method is based on analyzing the spectrum of the graph Laplacian in order to extract a feature vector for the classification algorithm. Firstly we calculate the normalized graph Laplacian

$$\begin{array}{c} L=I-D^{-1/2}AD^{-1/2} \end{array}$$ (3)

where *D* is the degree matrix, A is the adjacency matrix of the graph and I is the identity. The input into the classifier is then the *k* smallest eigenvalues of the Laplacian in ascending order

$$\begin{array}{c} X=(\lambda_{1},\lambda_{2},\ldots \lambda_{k}) \end{array}$$ (4)

These eigenvalues will be between 0 and 2, which is very convenient when using any classifier, as no re-scaling or normalization is required.

The inspiration for this method comes from an embedding method originally proposed by Belkin and Niyogi [38, 39], who demonstrated that using the eigenvectors corresponding to the smallest non-zero eigenvalues is equivalent to minimizing the distance between nodes in a lower dimension. However, most of the work in this field relates to the eigenvectors of the Laplacian rather than the eigenvalues. The presence of certain eigenvalues in the spectrum of the Laplacian is known to indicate certain structures [40], however this relationship is complex and not fully studied. Empirically this method shows good results in detecting network differences [32, 41].

We are not aware of an objective method of selecting *k*, but in this paper we set it to 20. In general, as long as *k* is set above 10, the results tend to be fairly similar.

Once we have an embedding for each graph we then use a random forest classifier to classify the networks. A random forest classifier works by fitting a set of decision trees on subsets of the data [42]. The resulting trees then vote on the class of new samples, and the class the majority vote for is taken. This sampling process allows the method to avoid overfitting the training set, and random forests are generally very good at classifying different types of data. Their downside is that the models produced are not very interpretable, but for our use in this paper, this is not an issue. We have taken a similar approach before, on word co-occurrence networks [43], with good results, and the authors in the original paper also obtained good results using a random forest classifier. We show results using logistic regression, and an SVM with a Linear and RBF kernel in the supplementary material.

To evaluate the resulting accuracy, we use the mean accuracy from 10 fold cross validation.

In this paper, we do two classification tasks. The first task is to classify correlation matrices drawn from the windows of highest and lowest volatility. The second is to classify correlation matrices drawn from the window with median volatility and the window adjacent to this. The idea here is to have two classification tasks of differing difficulties, as the first task should be significantly easier than the second. We draw 50 correlation matrices of each type.

The input into the SF method is then the sample correlation matrices, and then the resulting networks after the MST, PMFG and TMFG methods are run. As mentioned in the introduction, the motivation for running these filtration methods is to remove noisy edges in correlation matrices when we do not have many samples. Therefore, we vary the number of samples in each of the sub- datasets, running the procedure from *n* = 10 to *n* = 200. We also run the classification procedure for 4 different values of *p* for each dataset—*p* = 50, 100, 150, 200 for the US, *p* = 20, 40, 60, 70 for the UK, *p* = 5, 10, 15, 20 for Germany, *p* = 10, 20, 30, 40 for India and *p* = 10, 15, 20, 25 for China. We would expect that removing the noisy edges should improve classification accuracy when *n* is smaller.

For both of these classification tasks, we also calculate a correlation matrix using the Ledoit-Wolf shrinkage method [44]. Their model combines the identity matrix (*I*) and the sample covariance matrix (*S*) to produce a new estimate as follows

$$\begin{array}{c} S_{*}=\left(1-\rho \right)S+\rho \frac{tr(S)}{p}I \end{array}$$ (5)

where tr(*S*) is the trace of *S*. This reduces the off-diagonal values of the resulting correlation matrix to make it more well formed.

To decide *ρ* the authors wish to minimize the Frobenius norm of the difference between the estimated covariance matrix S_*_ and the true population covariance matrix Σ_*_

$$\begin{array}{c} \underset{\rho }{\text{min}}\;E[||\Sigma_{*}-S_{*}{\left|\right|}_{F}^{2}] \end{array}$$ (6)

The optimal solution of *ρ* is

$$\begin{array}{c} \rho =\frac{E[||S-\Sigma_{*}|{|}_{F}^{2}]}{E[||S-tr\left(S\right)I|{|}_{F}^{2}]}=\frac{\beta^{2}}{\delta^{2}} \end{array}$$ (7)

The interpretation here is that if *S* is very close to Σ_*_ (i.e. our estimate of the covariance is good) then we do not need to shrink much, or if our shrinkage choice is not very good then we should not shrink much either. However the obvious flaw so far is that we need to know the true population covariance matrix to obtain the correct value for *ρ*—and if we did then we would not need to bother estimating it to begin with! We therefore require estimates of *β*^2^ and *δ*^2^. We can estimate *δ*^2^ as following:

$$\begin{array}{c} \hat{\delta^{2}}=||S-{tr\left(S\right)I||}_{F}^{2} \end{array}$$ (8)

and *β*^2^ as

$$\begin{array}{c} \hat{\gamma^{2}}=\frac{1}{n^{2}}\sum_{k=1}^{n}\left|\right|x_{k}x_{k}^{T}-S{\left|\right|}_{F}^{2} \end{array}$$ (9)

$$\begin{array}{c} \hat{\beta^{2}}=\text{min}\left(\hat{\delta^{2}},\hat{\gamma }\right) \end{array}$$ (10)

$$\begin{array}{c} \hat{\rho }=\frac{\hat{\beta^{2}}}{\hat{\delta^{2}}} \end{array}$$ (11)

The constraint on $\hat{\beta^{2}}$ ensures that *ρ* < 1. While it is rarely necessary, it does help stop us accidentally making our estimate less well formed.

This resulting covariance matrix S_*_ can then be turned into a correlation matrix as follows

$$\begin{array}{c} C_{{*}_{ij}}=\frac{S_{{*}_{ij}}}{\sqrt{S_{{*}_{ii}}S_{{*}_{jj}}}} \end{array}$$ (12)

### 2.4 Software and data

The data we use is downloaded from Yahoo Finance. For the UK data we use the FTSE100 companies, for the US returns we use the S&P500 companies, for the German (DE) returns we use the DAX30 companies, for the Indian (IN) returns we use the NIFTY 50 and for the Chinese (CH) returns we use the SSE50. For the US, UK and Germany we use returns from 2000/03/01 to 2019/10/21. For the Indian and Chinese markets we use returns from 2008/01/01—2019/10/21. This is due to the large number of changes in the companies that make up these indices compared to those from the US, UK and Germany. For each dataset, any company missing more than 10% of its data is removed, and any missing values are filled forwards from the first good value.

This results in 5065 days of return data for 70 companies for the UK, 5068 days for 23 companies for Germany, 4790 days of return data for 229 companies for the US, 2903 days for 47 companies for India and 2855 days for 34 companies for China.

For these datasets we calculate the absolute Pearson correlation coefficient between the log returns.

We make use of Python, NumPy and SciPy [45] for general scripting, pandas [46] for handling the data, matplotlib [47] for plotting, Networkx [48] for the network analysis and TopCorr (https://github.com/shazzzm/topcorr) for constructing the filtered networks.

## 3 Results

### 3.1 Degree correlation

**Fig 2:**
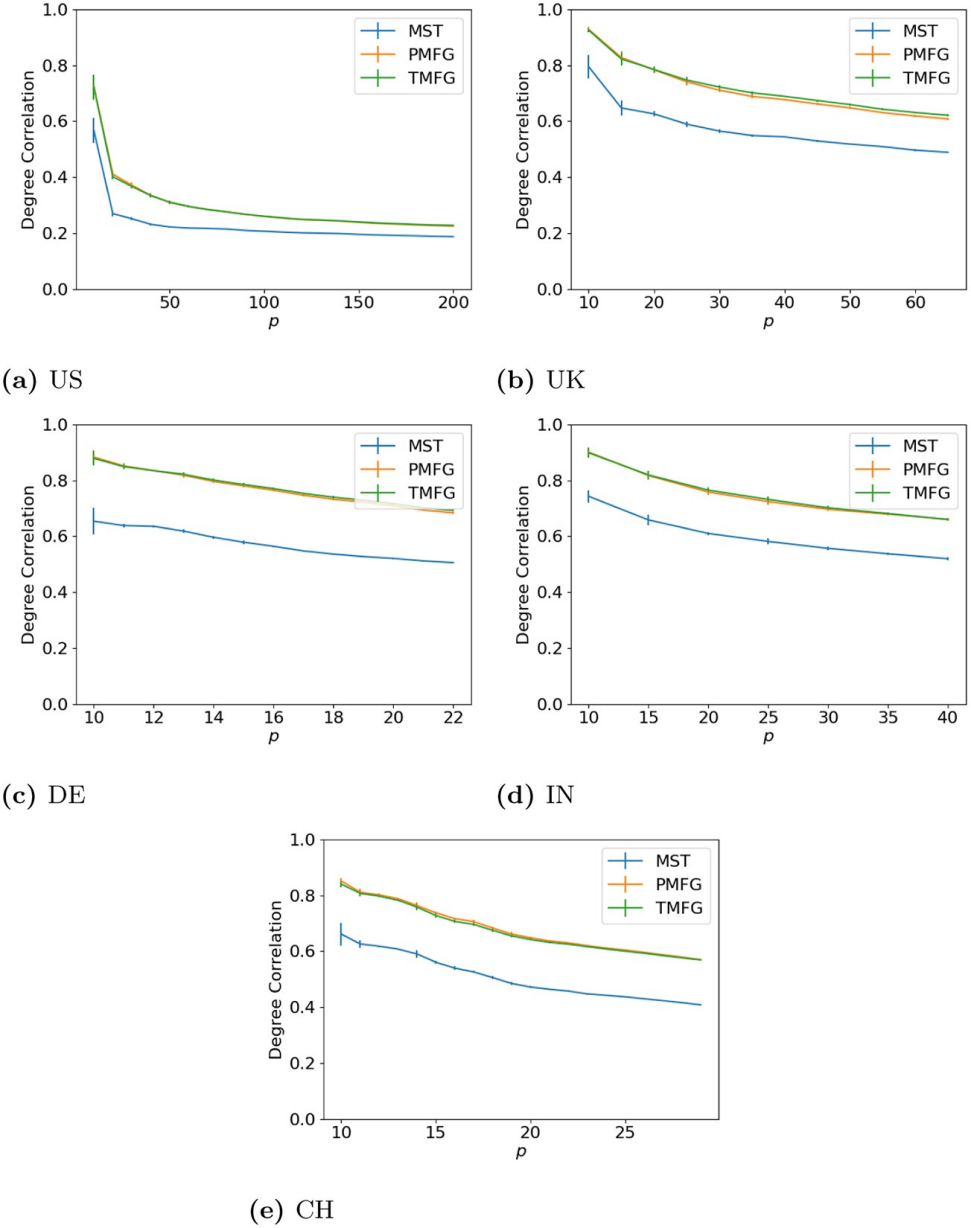
Degree agreement between the full and filtered correlation matrices with different values of *p* for the windows with maximum volatility. (**a**) US, (**b**) UK, (**c**) DE, (**d**) IN, (**e**) CH.

To start with we look at the effects of the filtration methods by comparing the weighted degree of a node in the full and filtered networks as we vary *p*. Our goal here is to show how the size of the resulting correlation matrix affects the relationship between the full and filtered networks. The results are shown in Figs 2 (maximum volatility), 3 (minimum volatility) and 4 (median volatility).

**Fig 3:**
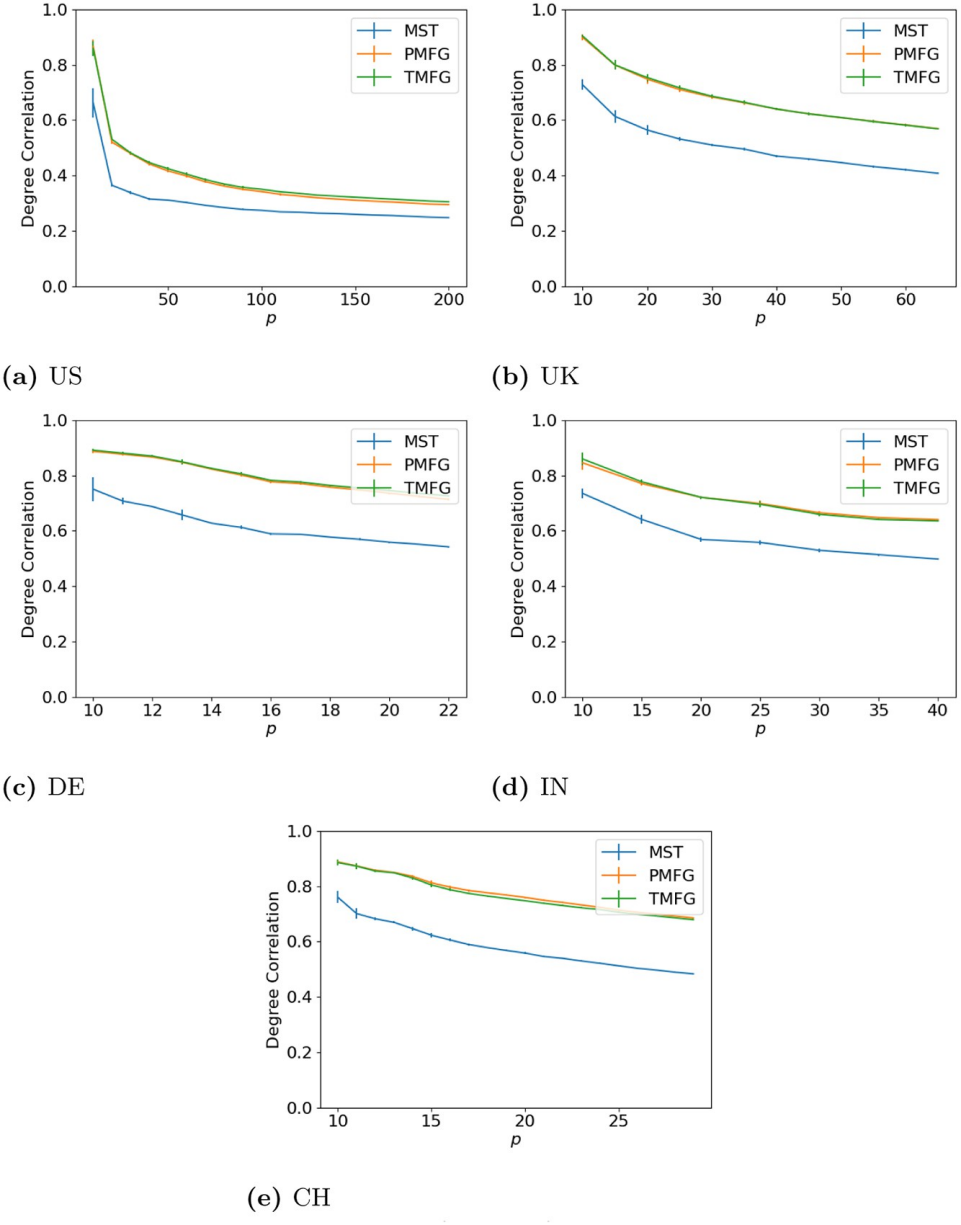
Degree agreement between the full and filtered correlation matrices with different values of *p* for the windows with minimum volatility. (**a**) US, (**b**) UK, (**c**) DE, (**d**) IN, (**e**) CH.

**Fig 4:**
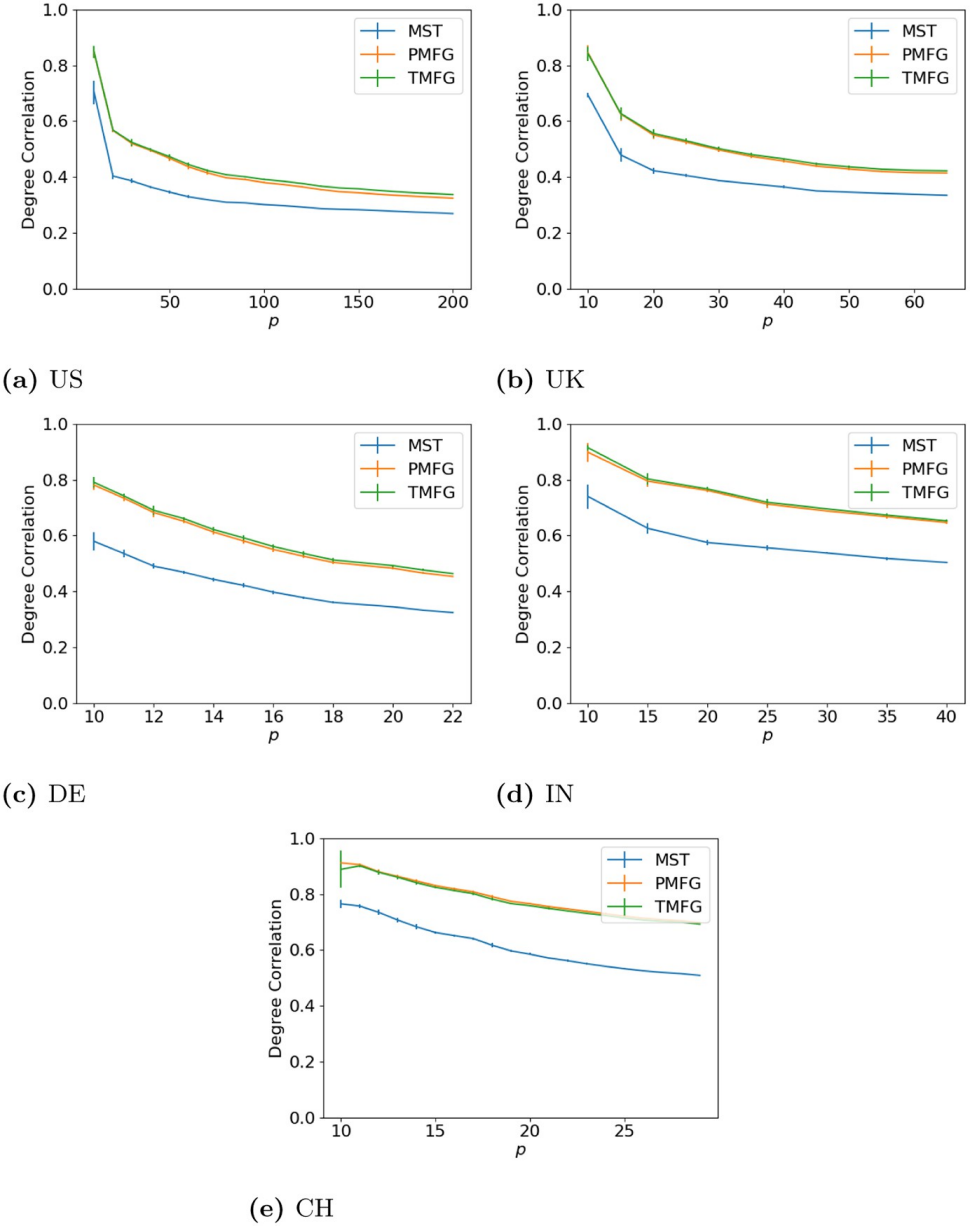
Degree agreement between the full and filtered correlation matrices with different values of *p* for the windows with median volatility. (**a**) US, (**b**) UK, (**c**) DE, (**d**) IN, (**e**) CH.

Over all of the datasets, the correlation drops as *p* increases. As we increase the number of nodes in the correlation networks, we discard more correlations (as the number of edges in the full networks is *p*(*p* − 1), but only 3*p* − 6 in the PMFG and TMFGs and by *p* − 1 in the MSTs.), hence why this occurs. We can also see how the network size affects this—the larger US market allows the correlation to drop much lower than the smaller markets from the other countries. The shape for all three market states is similar between all countries, indicating this relationship is relatively stable, and does not seem to change with market type either (i.e. developing vs developed).

### 3.2 Edge correlation

**Fig 5:**
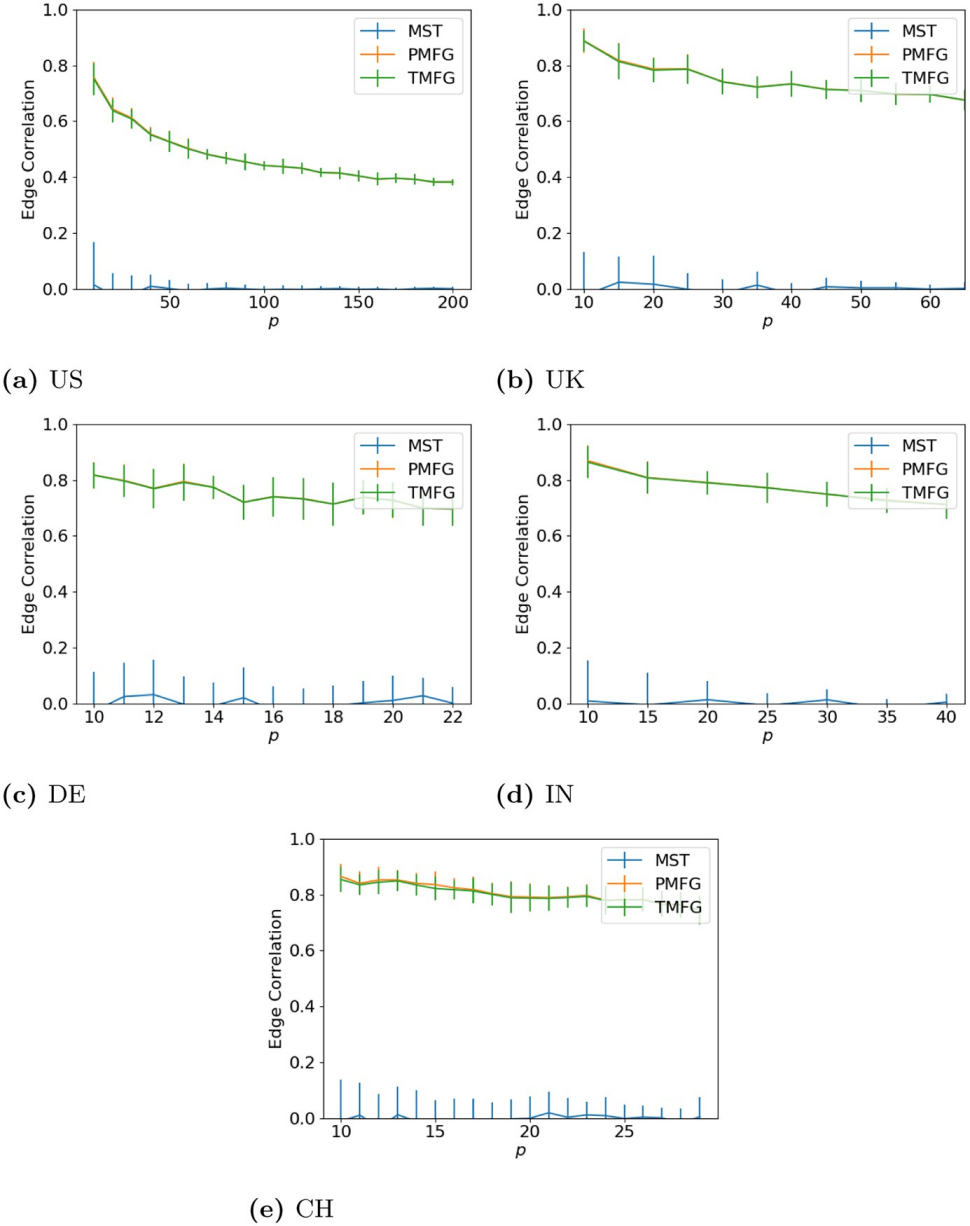
Agreement between edges between the full and filtered correlation matrices with different values of *p* for the window with maximum volatility. (a) US, (b) UK, (c) DE, (d) IN, (e) CH.

Here we look at how the edges differ between the full and filtered networks. Looking at the results for this (Figs 5 (maximum volatility), 6 (minimum volatility) and 7 (median volatility) we can see that there is almost no relationship between the MST and full correlation networks for any data set, while the PMFG and TMFG show much stronger relationships. As with the degree correlation above, the edge correlation drops as we increase *p* and discard more correlations. Again the shape of the line is similar for every market state and type, with the developed and developing markets looking similar.

**Fig 6:**
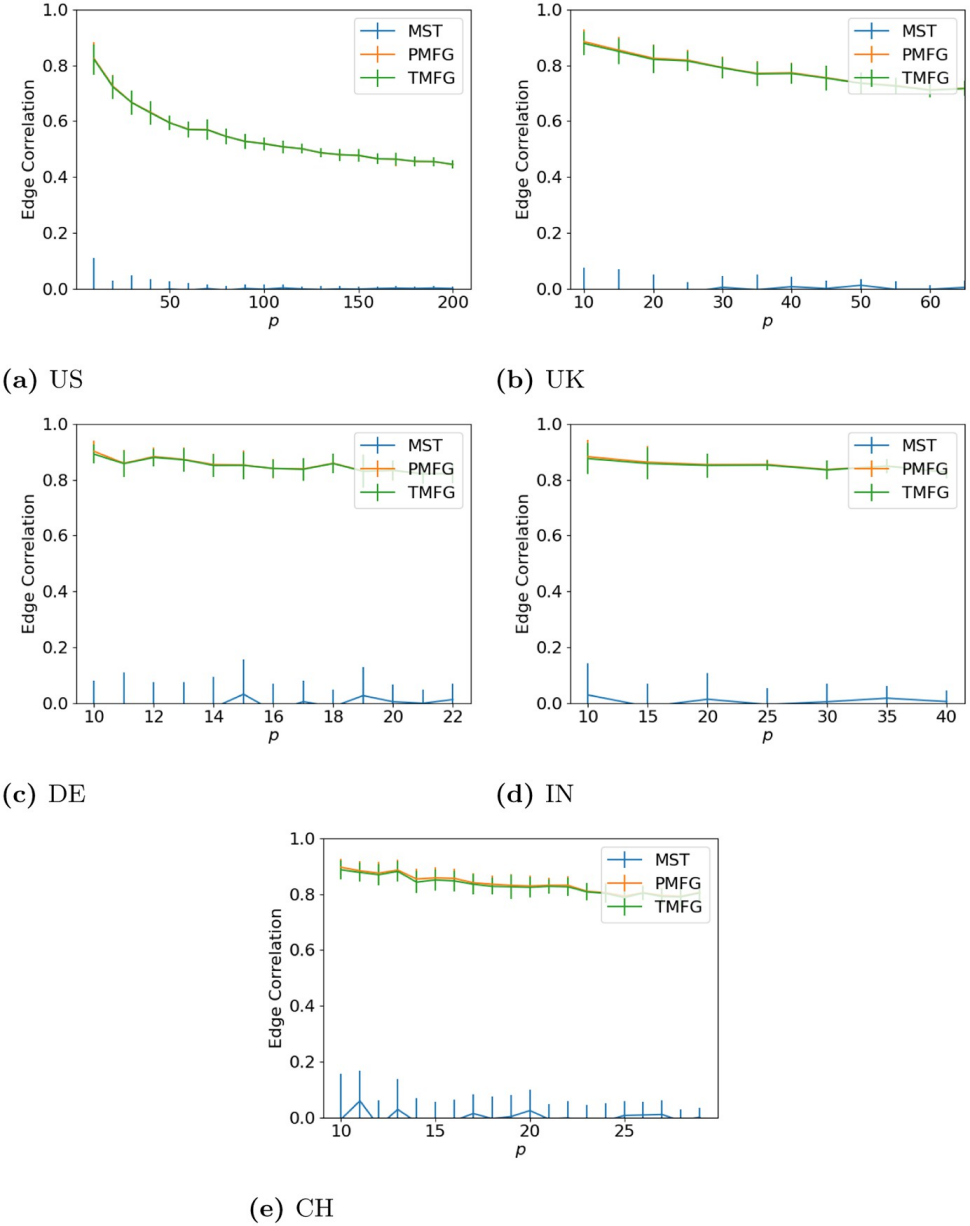
Agreement between edges between the full and filtered correlation matrices with different values of *p* for the window with lowest volatility. (a) US, (b) UK, (c) DE, (d) IN, (e) CH.

**Fig 7:**
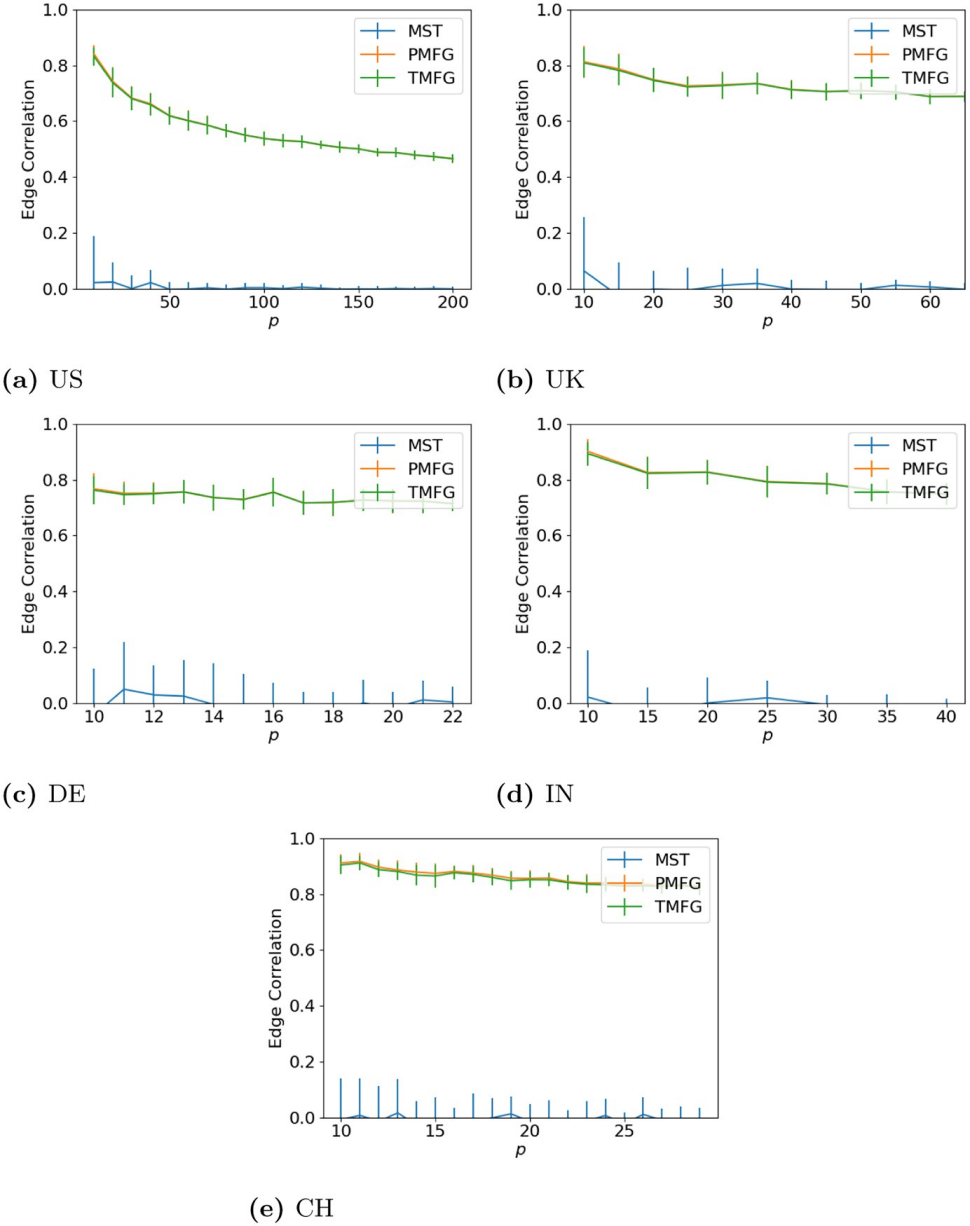
Agreement between edges between the full and filtered correlation matrices with different values of *p* for the window with median volatility. (a) US, (b) UK, (c) DE, (d) IN, (e) CH.

### 3.3 Variation over time

**Fig 8:**
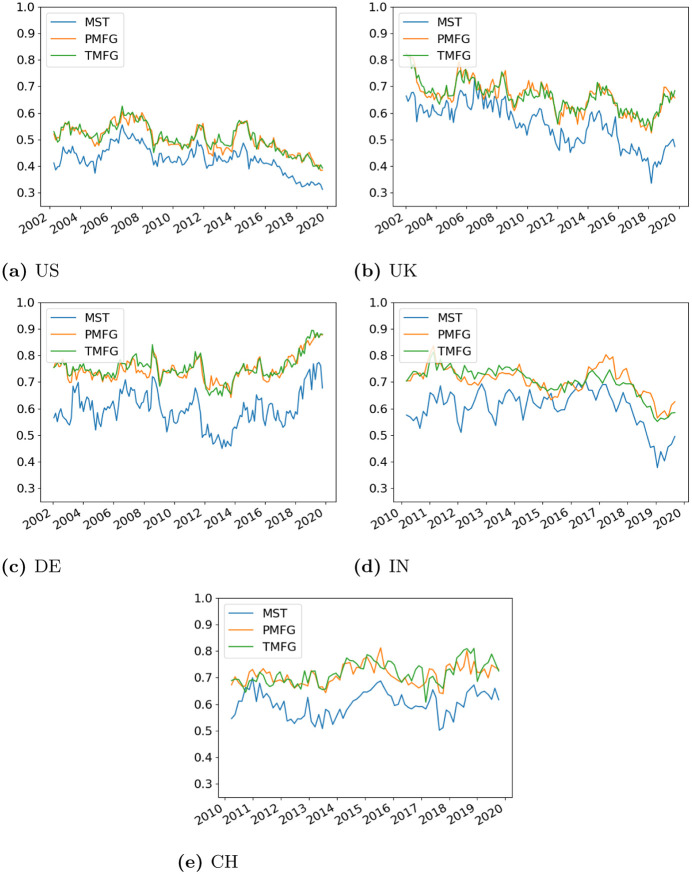
Correlation between degree centrality in the full and filtered networks over the dataset. (a) US, (b) UK, (c) DE, (d) IN, (e) CH.

**Fig 9:**
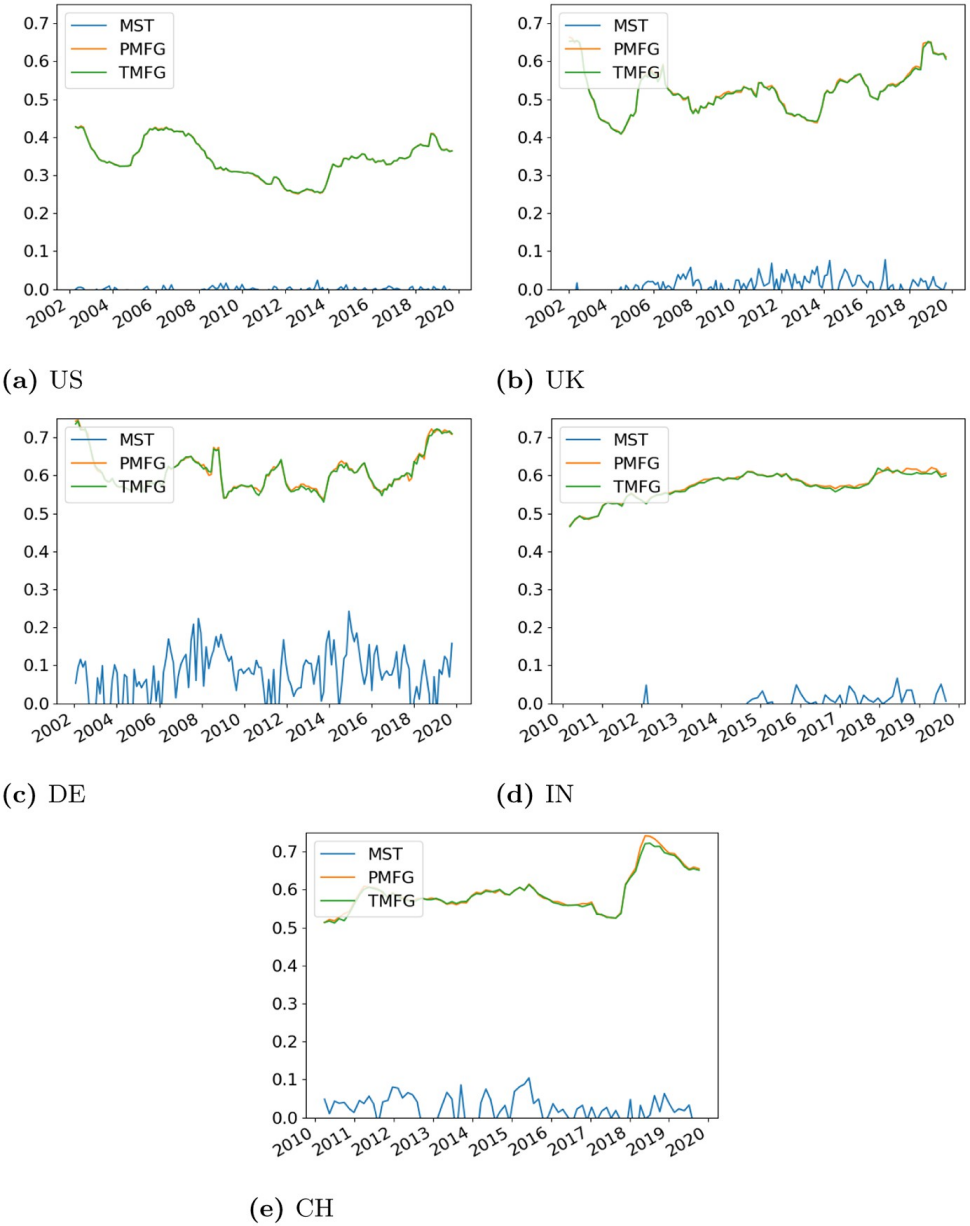
Edge agreement between the full and filtered networks over the time period of the dataset. (a) US, (b) UK, (c) DE, (d) IN, (e) CH.

Having explored how the agreements vary over the dimensionality of the data, next we look at how they vary over time throughout the dataset. The degree centrality correlation over time is shown in Fig 8 and the edge centrality agreement is shown in Fig 9.

For the degree centrality agreement, again the PMFG and TMFG generally have very similar trends, while the MST has a lower agreement. Having said that, bearing in mind how many more edges the MST discards compared to the other two methods, we note that the degree centrality agreement is still quite high over the entire dataset. There is quite a large amount of variation over time, notably for the smaller UK and German markets.

Next we look at edge agreement (Fig 9). The PMFG and TMFG show a similar level of agreement with the unfiltered networks. However, the sparsity of the MST procedure becomes very noticeable, with the edge agreement being very low across all countries. This measure is much less variable over time than the degree correlation.

Our goal in this section was to quantify whether these measures vary with the state of the market. From the figures alone, it is difficult to see any obvious pattern, and so we look at how the measures are related to the volatility of the market. Again this is defined using the mean of the sum of the variances of each asset in the window, and we use Spearman correlation to measure the relationship between volatility and the degree/edge agreements. The results of this are shown in Table 1.

**Table 1:** Spearman correlation between the degree and edge agreement measures and the volatility of the market. Correlations insignificant at *p* < 0.05 are in italic.

	Filtration Method	US	UK	DE	IN	CH
Degree Agreement	MST	*-0.012*	0.683	-0.108	0.217	*0.173*
	PMFG	*-0.022*	0.437	-0.302	0.423	-0.348
	TMFG	*-0.027*	0.523	-0.232	0.636	-0.322
Edge Agreement	MST	*0.107*	-0.403	-0.265	-0.608	*0.052*
	PMFG	-0.494	-0.262	-0.256	-0.801	-0.619
	TMFG	-0.494	-0.264	-0.235	-0.771	-0.649

For the degree agreement, we see each country having a different relationship, with the US having none, the UK and India having more degree agreement as the markets become more volatile, while China and Germany having a negative relationship.

For the edge agreement, we see that most countries have a negative relationship between agreement and volatility, indicating that the filtered and full correlation networks agree with each other more when the networks are less volatile. The developing markets have a stronger correlation than the developed markets. This seems quite reasonable, as in general during times of stress the strength of correlations increases [7, 49]. Therefore we will be discarding larger correlations during times of stress compared to calm with the filtration methods. Developing markets also have larger correlations compared to developed ones [27]. However the strength of the relationship varies strongly across the different countries and filtration methods—the MST shows little correlation for the US and China, but strong negative correlation for India.

### 3.4 Graph classification

**Fig 10:**
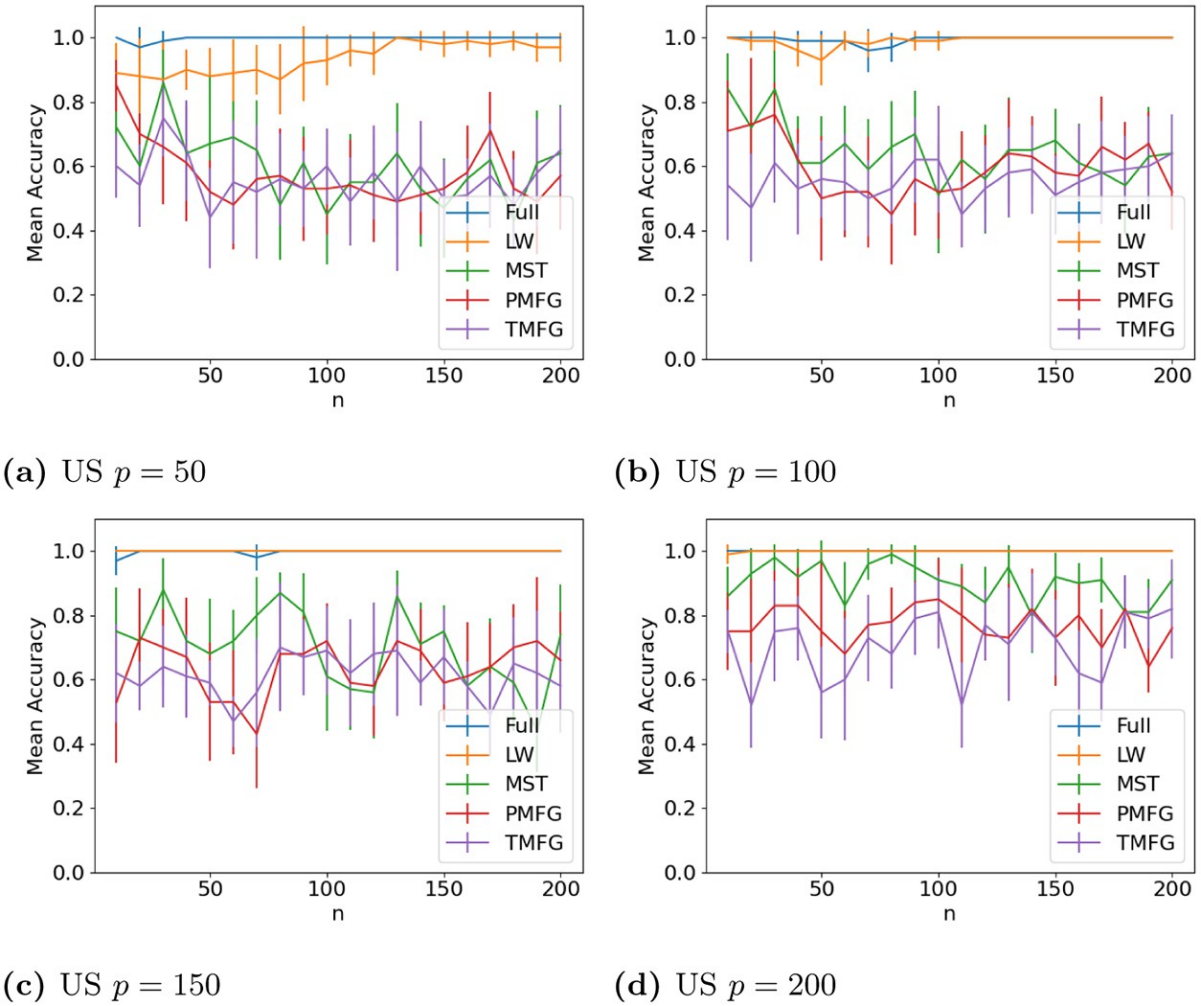
Accuracy when distinguishing between correlation matrices created from the stock windows with the maximum and minimum volatility using the SF method and a random forest classifier with US stock returns. (a) US *p* = 50, (b) US *p* = 100, (c) US *p* = 150, (d) US *p* = 200.

The results for classifying the networks from the maximum and minimum windows are shown in Figs 10 (US), 11 (UK), 12 (DE), 13 (India) and 14 (China), and for the windows adjacent to each other (also referred to as median) in Figs 15 (US), 16 (UK), 17 (DE), 18 (India) and 19 (China). Results for other classifiers can be found in the supplementary material.

**Fig 11:**
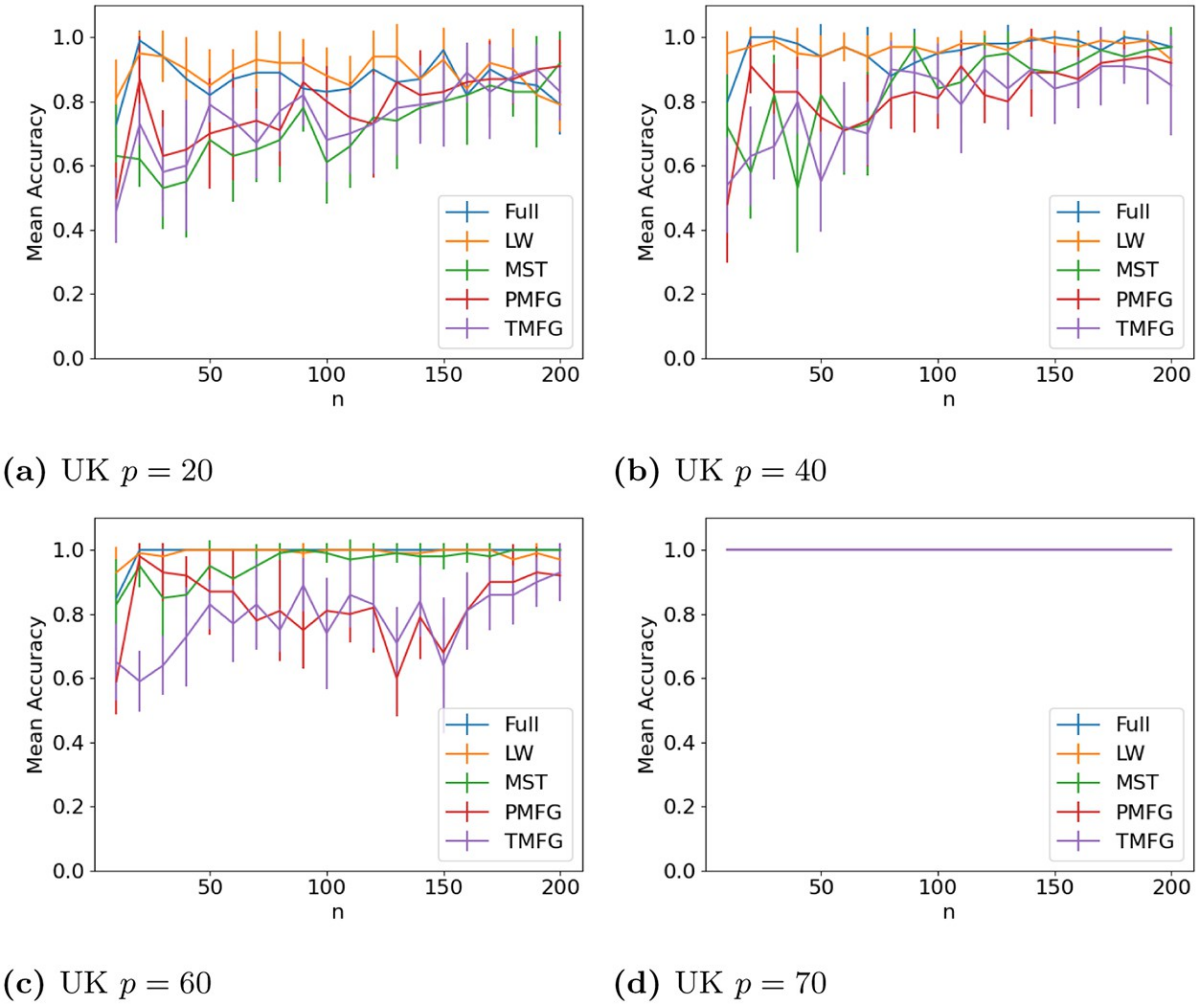
Accuracy when distinguishing between correlation matrices created from the stock windows with the maximum and minimum volatility using the SF method and a random forest classifier for UK stock returns. (a) UK *p* = 20, (b) UK *p* = 40, (c) UK *p* = 60, (d) UK *p* = 70.

**Fig 12:**
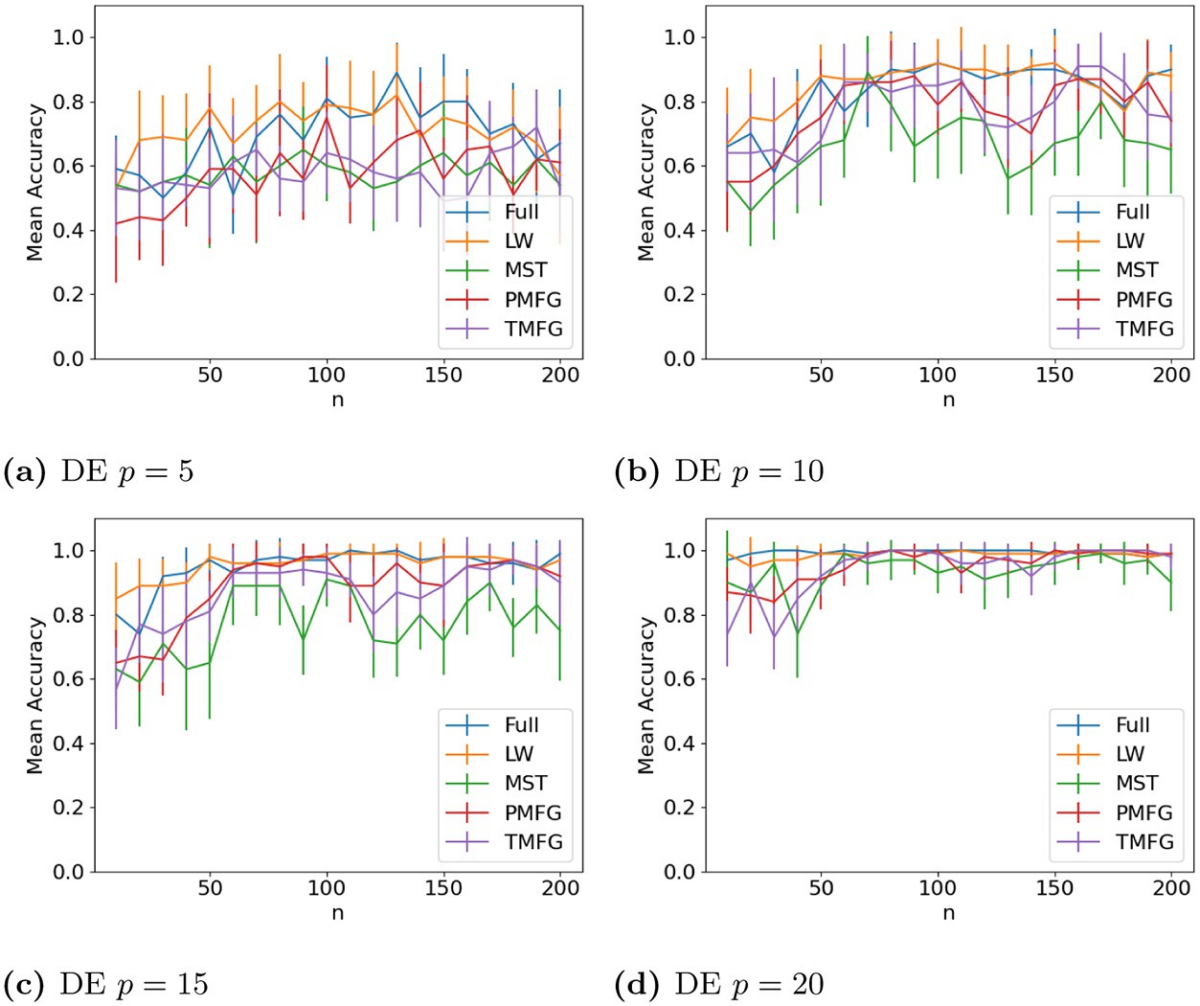
Accuracy when distinguishing between correlation matrices created from the stock windows with the maximum and minimum volatility using the SF method and a random forest classifier for Germany. (a) DE *p* = 5, (b) DE *p* = 10, (c) DE *p* = 15, (d) DE *p* = 20.

**Fig 13:**
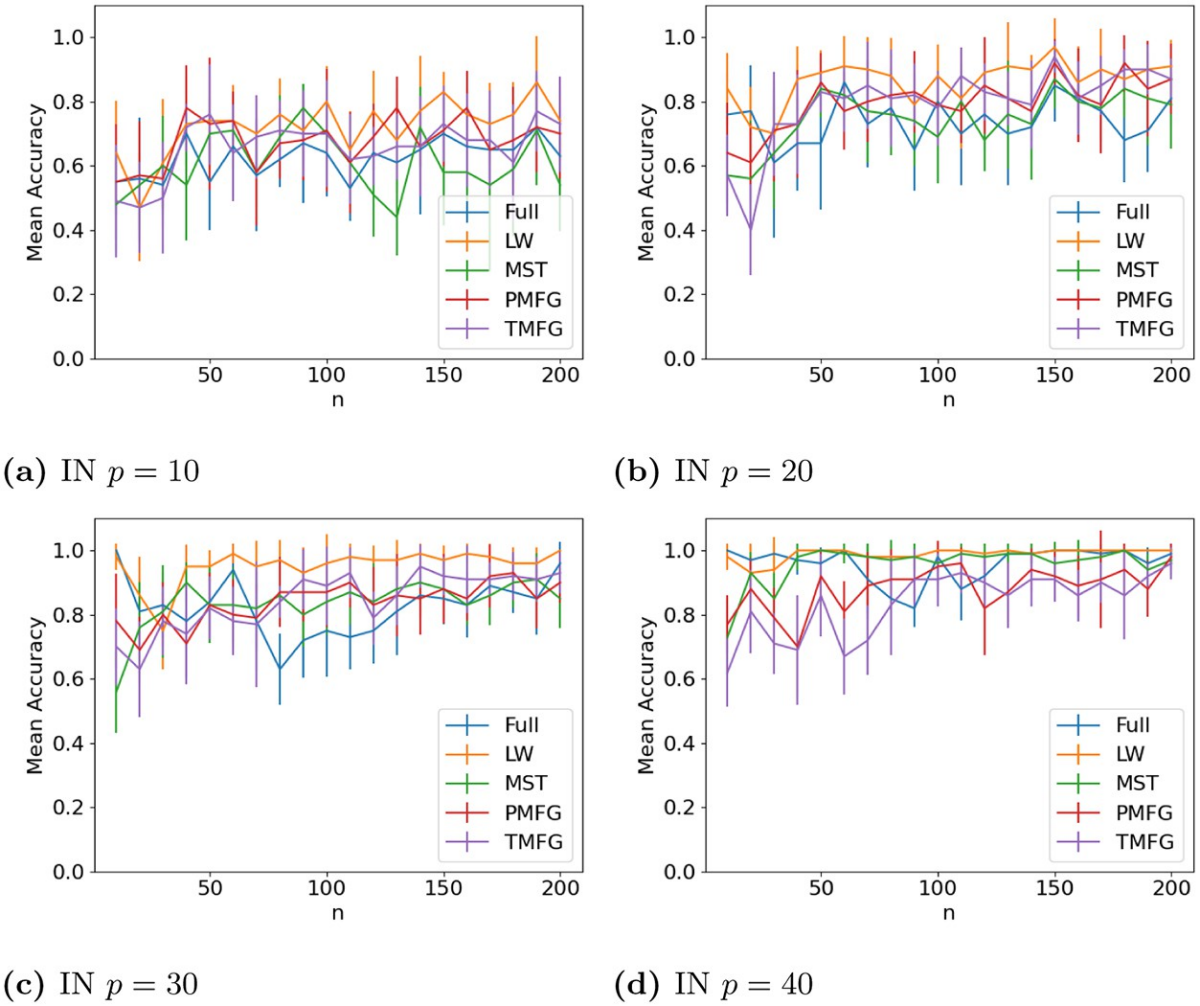
Accuracy when distinguishing between correlation matrices created from the stock windows with the maximum and minimum volatility using the SF method and a random forest classifier for India. (a) IN *p* = 10, (b) IN *p* = 20, (c) IN *p* = 30, (d) IN *p* = 40.

**Fig 14:**
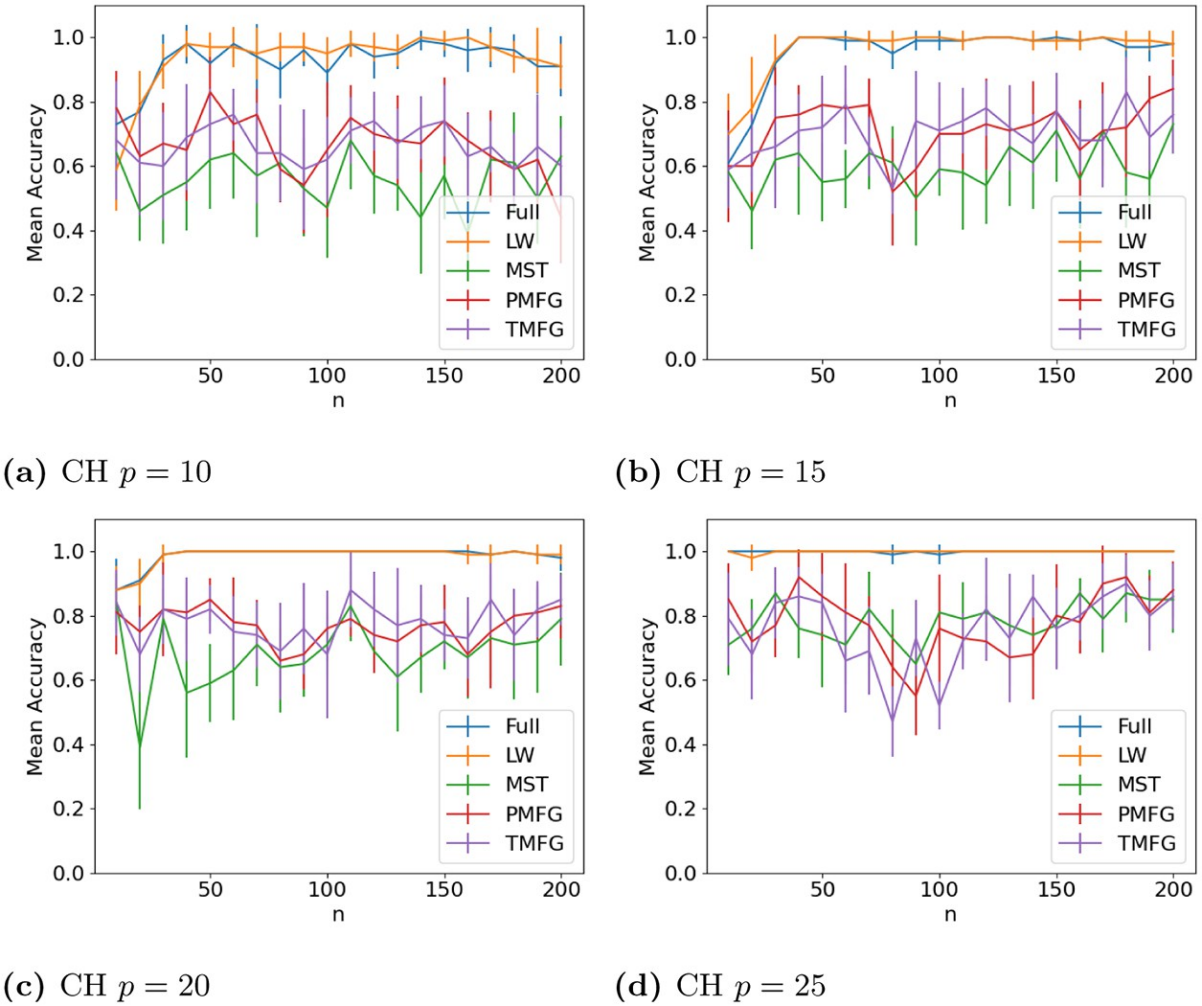
Accuracy when distinguishing between correlation matrices created from the stock windows with the maximum and minimum volatility using the SF method and a random forest classifier for China. (a) CH *p* = 10, (b) CH *p* = 15, (c) CH *p* = 20, (d) CH *p* = 25.

**Fig 15:**
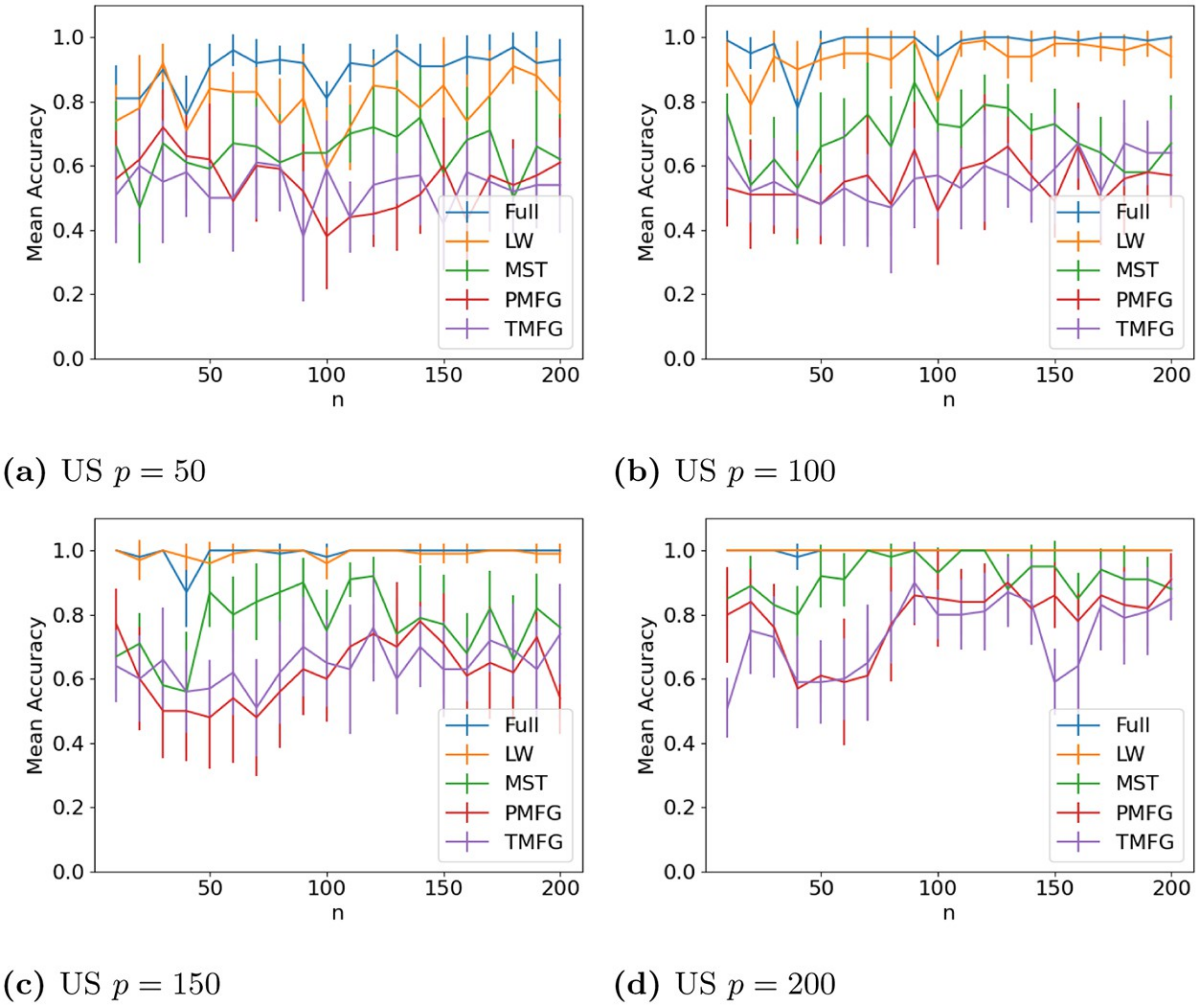
Accuracy when distinguishing between correlation matrices inferred from adjacent time windows based on the median volatility using the SF method and a random forest classifier for US stock returns. (a) US *p* = 50, (b) US *p* = 100, (c) US *p* = 150, (d) US *p* = 200.

**Fig 16:**
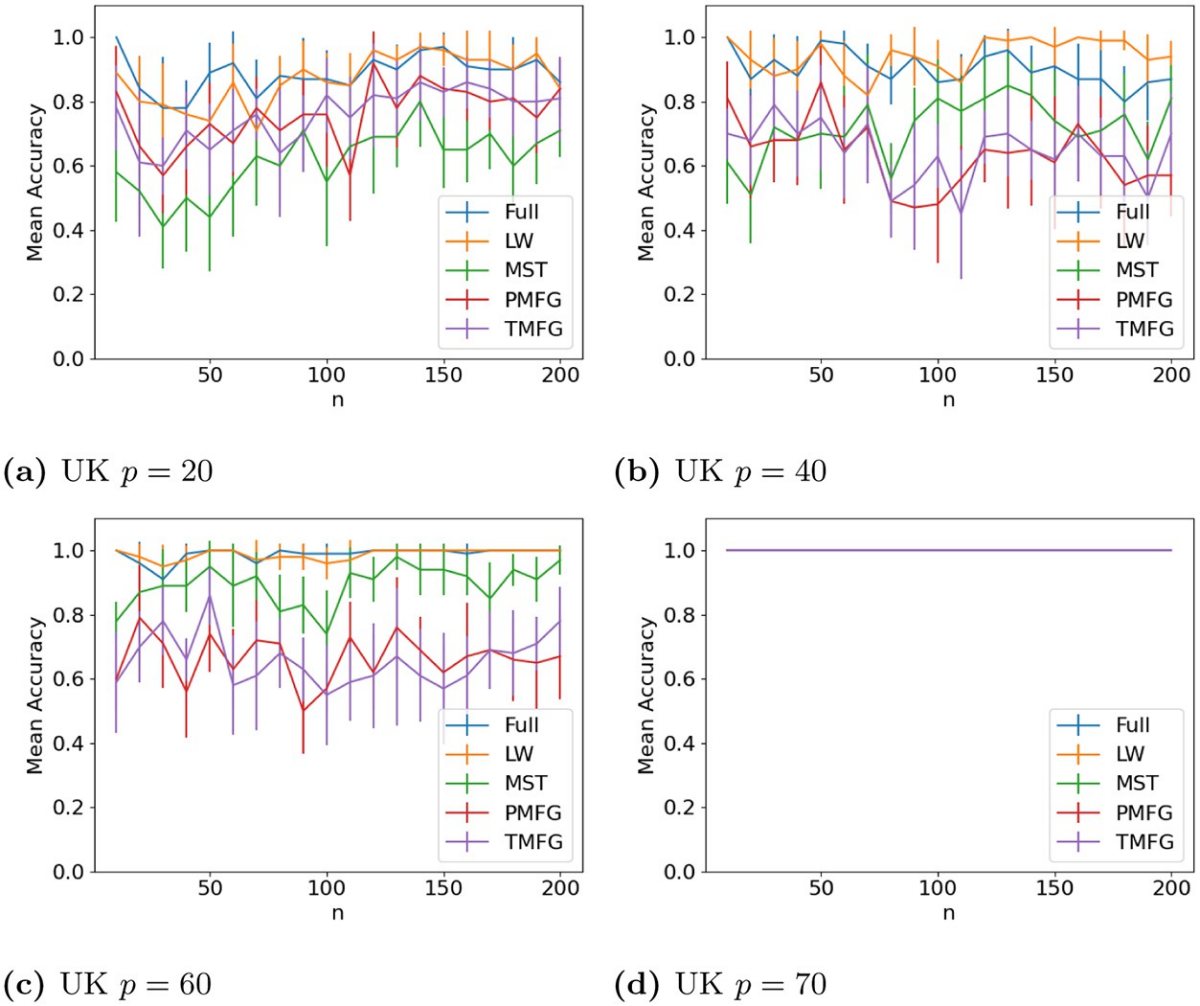
Accuracy when distinguishing between correlation matrices inferred from adjacent time windows based on the median volatility using the SF method and a random forest classifier for UK stock returns. (a) UK *p* = 20, (b) UK *p* = 40, (c) UK *p* = 60, (d) UK *p* = 70.

**Fig 17:**
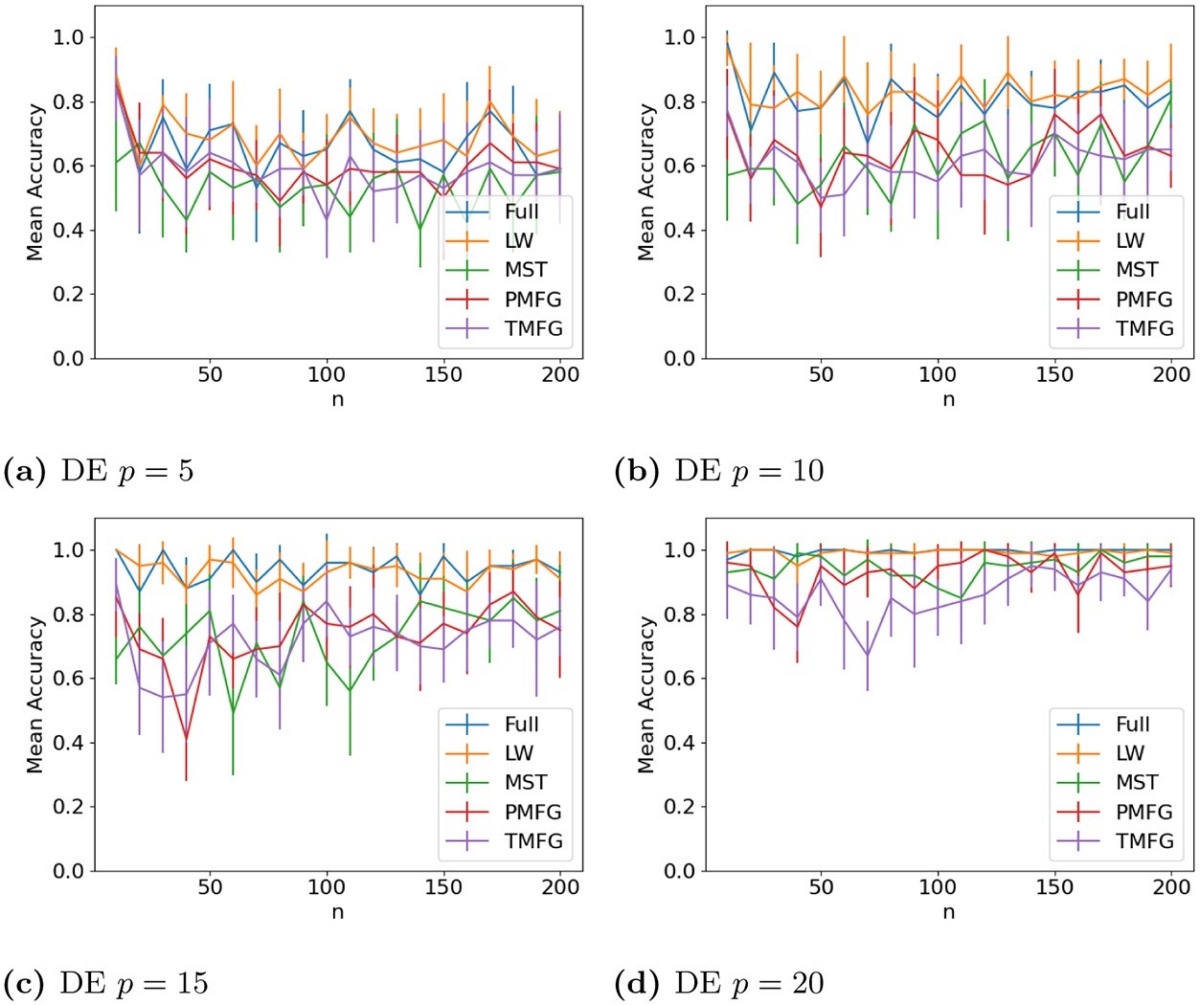
Accuracy when distinguishing between correlation matrices inferred from adjacent time windows based on the median volatility using the SF method and a random forest classifier for German stock returns. (a) DE *p* = 5, (b) DE *p* = 10, (c) DE *p* = 15, (d) DE *p* = 20.

**Fig 18:**
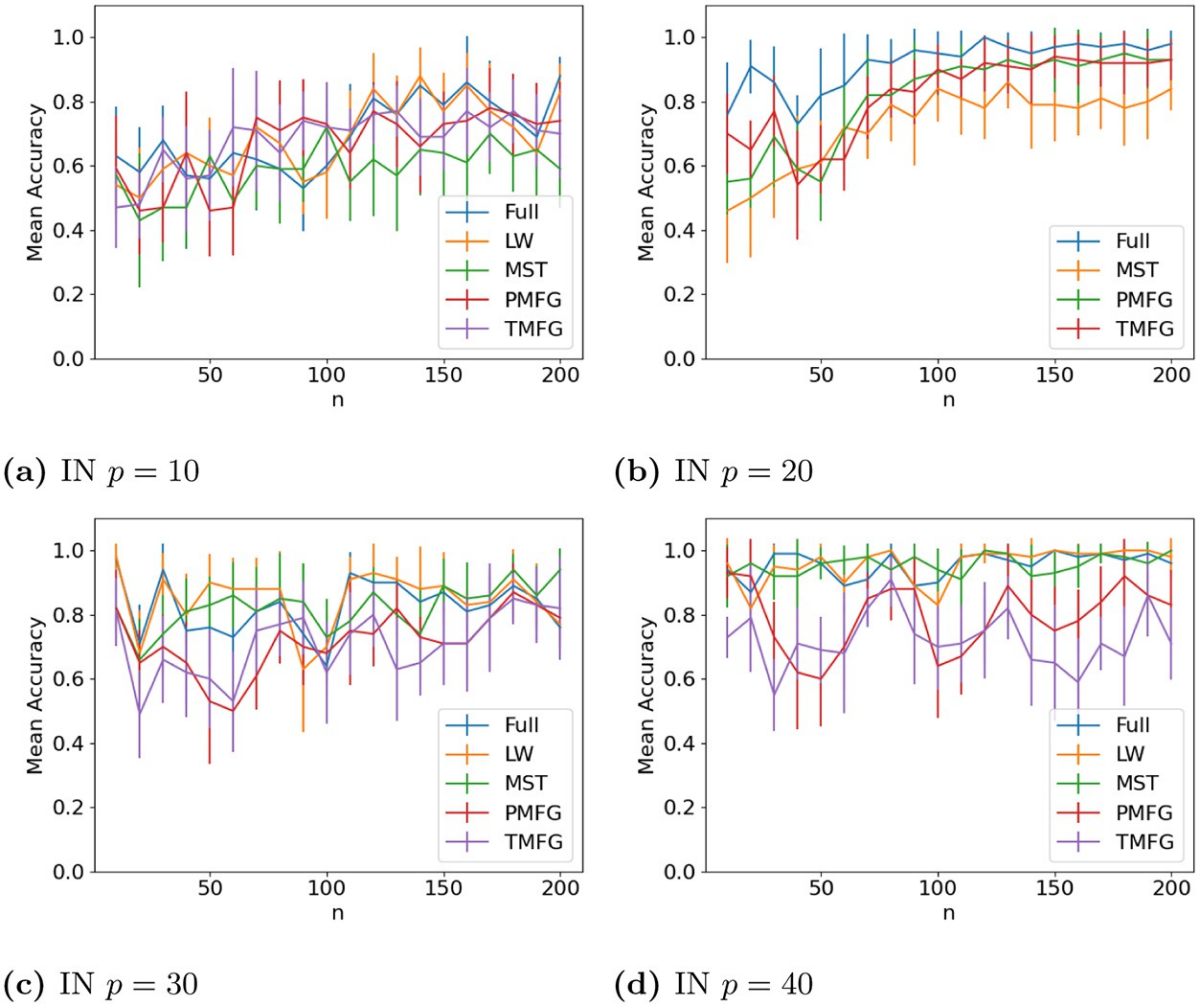
Accuracy when distinguishing between correlation matrices inferred from adjacent time windows based on the median volatility using the SF method and a random forest classifier for Indian stock returns. (a) IN *p* = 10, (b) IN *p* = 20, (c) IN *p* = 30, (d) IN *p* = 40.

**Fig 19:**
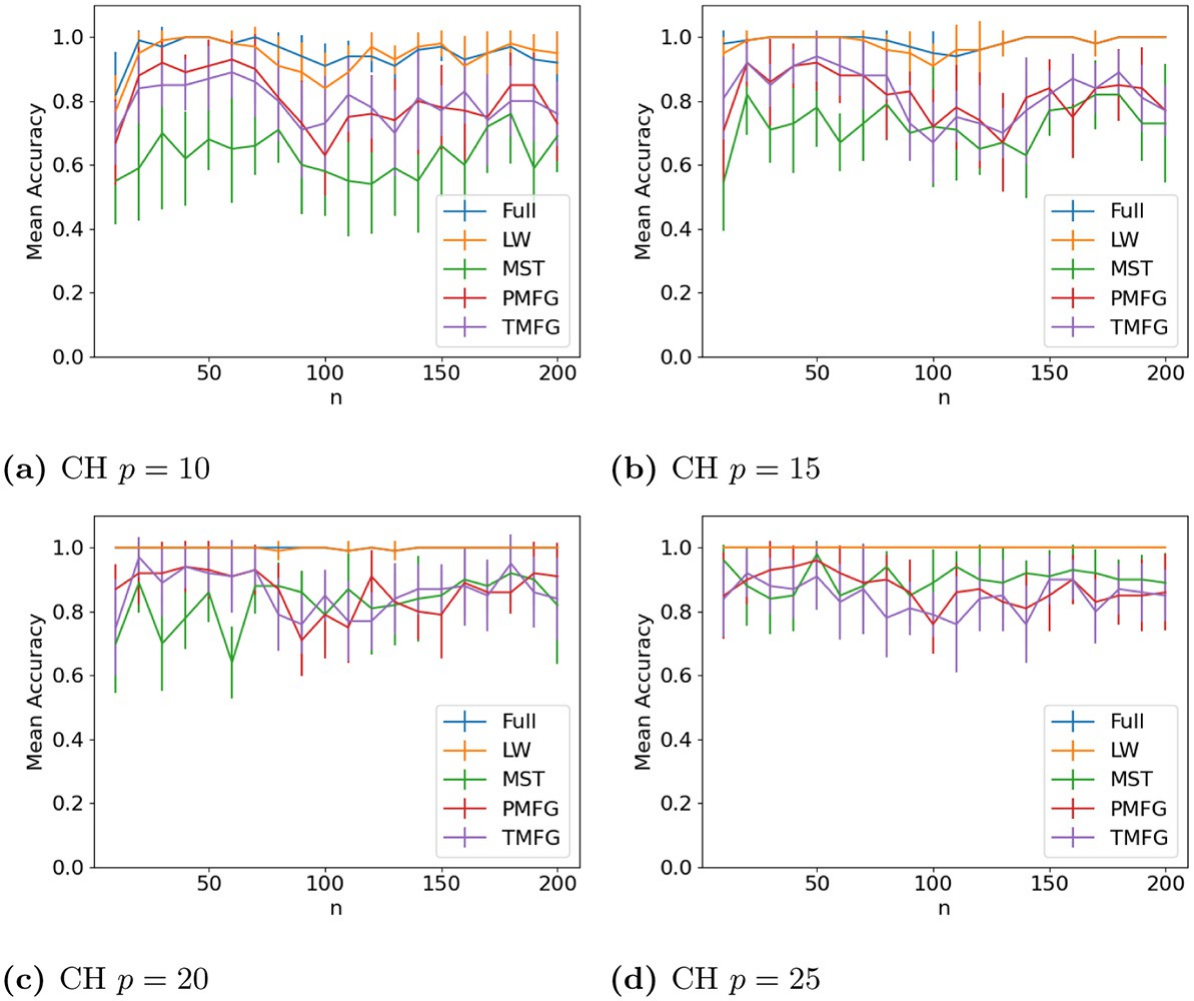
Accuracy when distinguishing between correlation matrices inferred from adjacent time windows based on the median volatility using the SF method and a random forest classifier for Chinese stock returns. (a) CH *p* = 10, (b) CH *p* = 15, (c) CH *p* = 20, (d) CH *p* = 25.

It is mostly possible to achieve high accuracy for both classification tasks, and for a few country/*p* combinations all methods can perfectly classify the test sets (notably the UK for *p* = 70). It is slightly easier to distinguish between the maximum and minimum windows than the adjacent ones, but the difference is not large. Classification accuracy also generally increases as *p* increases, and as the number of samples increases. There is also little difference between the developed (UK, US, DE) and developing markets (CH, IN) once the size of the correlation matrices is taken into account.

Surprisingly, we do not find that any of the filtration methods improve classification accuracy for any of the datasets when compared to the full correlation matrices. This indicates that the filtration methods are discarding edges which do contain information. If we compare the filtration methods, it seems that the MST has the highest accuracy when *p* is greater than 20. This is actually quite surprising, as we can see that just maintaining all of the edges seems to perform the best. From this we would therefore expect the methods that retain more edges to have a higher classification accuracy, rather than the MST, which discards a large number of edges. We did find that the MST had larger differences in edge structure between the two windows compared to the PMFG and TMFG, which would explain why the classification accuracy is higher, but this does not explain why the full networks performed the best. This could however be due to the large number of extra edges present, which makes up for the amount of noise.

Interestingly the Ledoit-Wolf shrinkage and unaltered correlation matrices show very similar results too. This would indicate that reducing the off-diagonal values is less useful in this task compared to in portfolio optimization, which the Ledoit-Wolf covariance tends to perform very well in.

These results hold up for most of the other classifiers too. Generally the random forest classifier has the highest accuracy, with the SVM with an RBF kernel second. Again we see a larger *p* helping improve classification accuracy. However with these alternative classifiers there is no filtration method which clearly performs the best.

## 4 Discussion and conclusion

In this paper we have investigated the effects of the three most used correlation matrix filtration methods using stock returns from five countries and compared their performance on a graph classification task. Firstly we find that the filtered networks do not always resemble the original full networks, and that this is dependent on the data, the dimensionality and the method. Unsurprisingly, the more edges that are discarded by the filtration method, the less the resulting network resembles the original. Furthermore, as we increase the number of dimensions, the filtered networks show less resemblance to the original networks, as more edges are being thrown away. While this may not be unexpected, it is worth bearing in mind when using these methods.

We also find that the agreement between the full and filtered networks varies over time, with the agreement on edge structure being negatively correlated to the market state. The agreement on degree centrality does not show as much of a consistent pattern, with positive, negative and no correlation being found for the five countries in our dataset.

In a more surprising result, we find that the correlation filtration methods do not seem to improve performance for most combinations of *p* and *n* on this task for any of the datasets when compared to the full correlation matrix. Comparing the methods, the MST seems to perform the best out of the three methods, and the PMFG and TMFG have very similar performances.

Due to this, we feel that future authors should be aware of the pitfalls of these methods—while they are convenient for producing networks that are easy to analyze, they may not necessarily produce a network that is closer to the truth. We would also encourage more future work on obtaining objective results comparing these methods. This could involve more and/or larger datasets than the ones considered here, or tasks other than classification (for instance portfolio optimization), or a different approach to classification. For instance, a study using graph distances such as in [50] to compare the filtration methods could be very interesting. We would also encourage similar work to this, but with higher frequency returns, which unfortunately were not available to us.

Finally, our results also show that the TMFG and PMFG have very similar performance. Since the TMFG is a more efficient algorithm (as it avoids the planarity check at every iteration), it should be worth using over the PMFG in most cases.

## Supporting information

S1 File (PDF)
