# Supplementary material for: An investigation into the effects and effectiveness of correlation network filtration methods with financial returns
Source: PLoS One. 2022 Sep 7;17(9):e0273830. doi: 10.1371/journal.pone.0273830 (PMC9451073; doi:10.1371/journal.pone.0273830)
Supplement: S1 File — (PDF) [file pone.0273830.s001.pdf]

Supplementary Material

Tristan Millington

July 13, 2022

1 Graph Classification with Alternative Classifiers

1.1 Germany

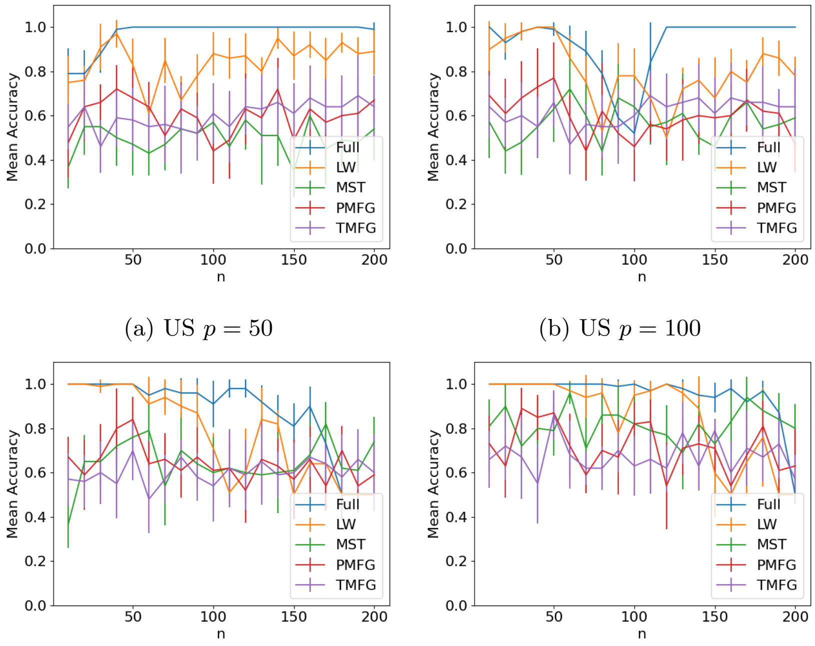

(a) US $p = 50$

(b) US $p = 100$

(c) US $p = 150$

(d) US $p = 200$

Figure 1: Logistic Regression - Max/Min

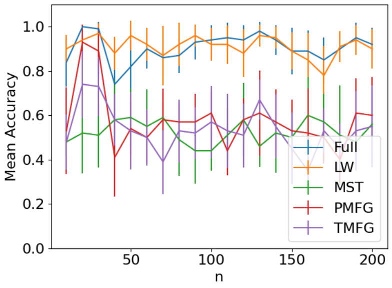

(a) US $p = 50$

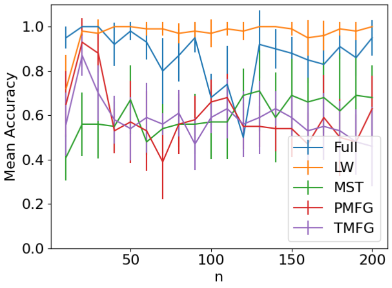

(b) US $p = 100$

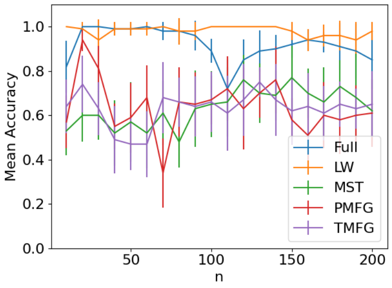

(c) US $p = 150$

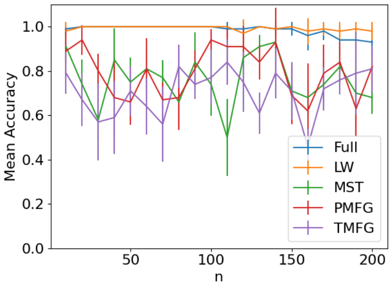

(d) US $p = 200$

Figure 2: Logistic Regression - Median

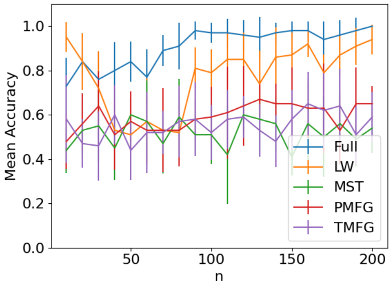

(a) US $p = 50$

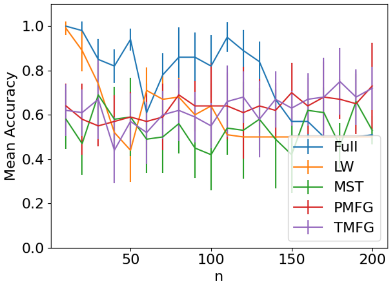

(b) US $p = 100$

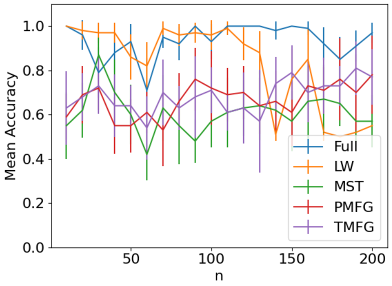

(c) US $p = 150$

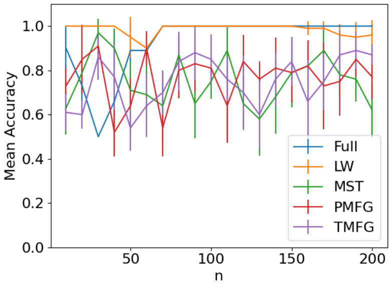

(d) US $p = 200$

Figure 3: Linear SVM - Max/Min

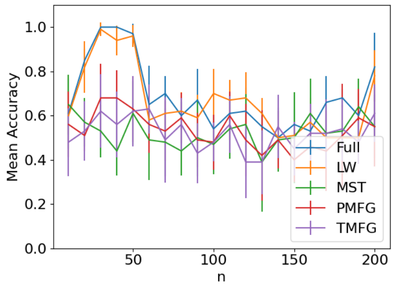

(a) US $p = 50$

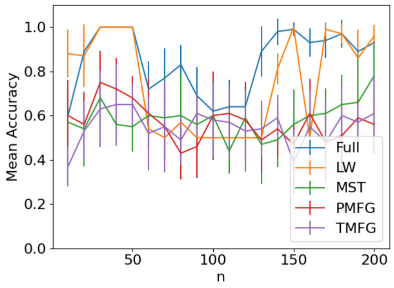

(b) US $p = 100$

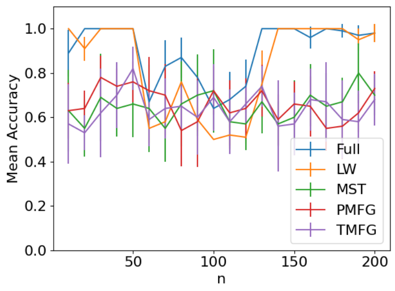

(c) US $p = 150$

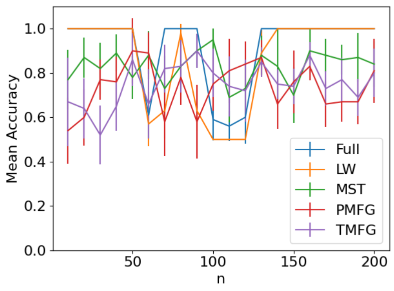

(d) US $p = 200$

Figure 4: Linear SVM - Median

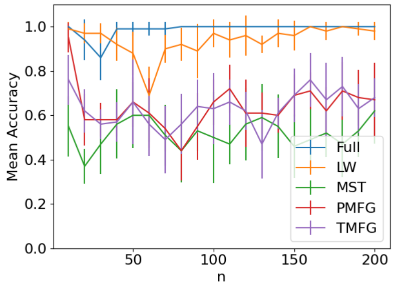

(a) US $p = 50$

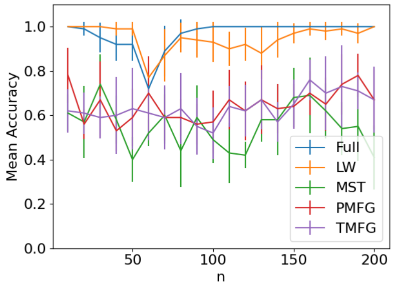

(b) US $p = 100$

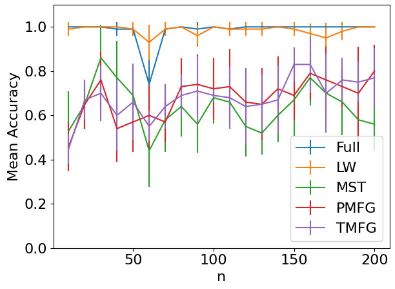

(c) US $p = 150$

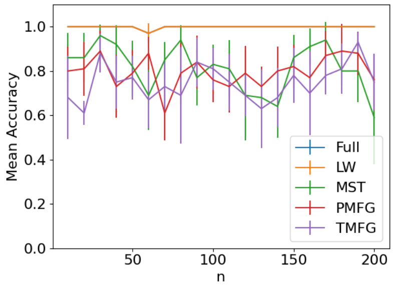

(d) US $p = 200$

Figure 5: RBF SVM - Max/Min

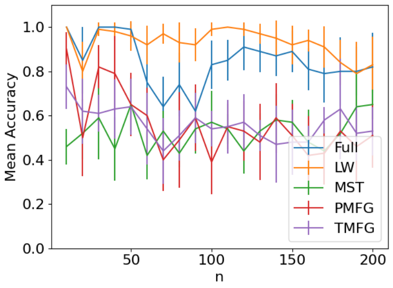

(a) US $p = 50$

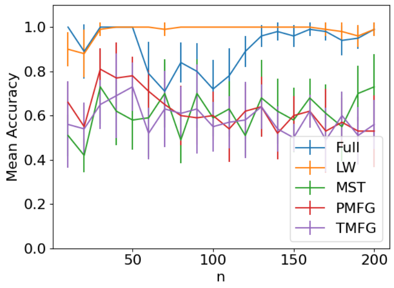

(b) US $p = 100$

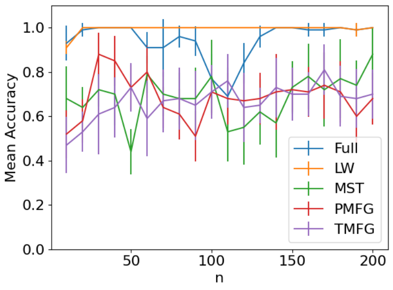

(c) US $p = 150$

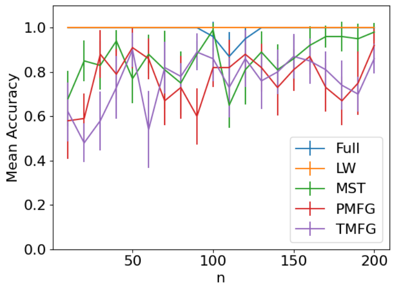

(d) US $p = 200$

Figure 6: RBF SVM - Median

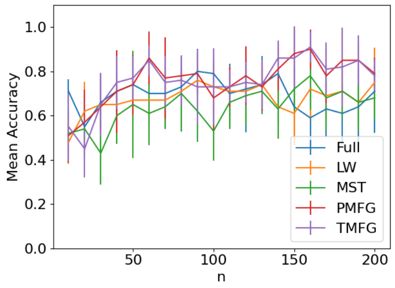

(a) UK $p = 20$

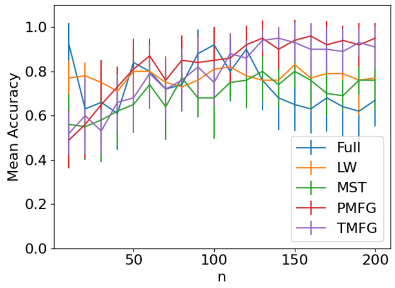

(b) UK $p = 40$

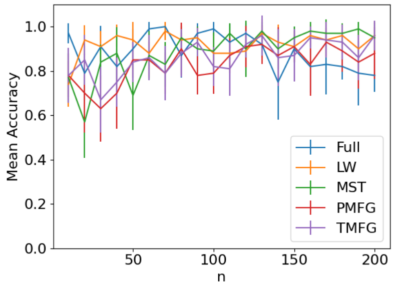

(c) UK $p = 60$

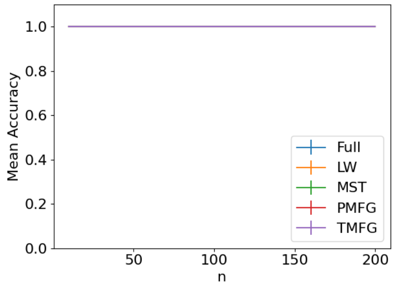

(d) UK $p = 70$

Figure 7: Logistic Regression - Max/Min

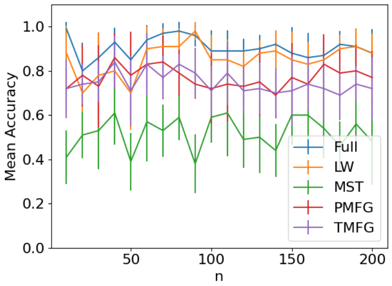

(a) UK $p = 20$

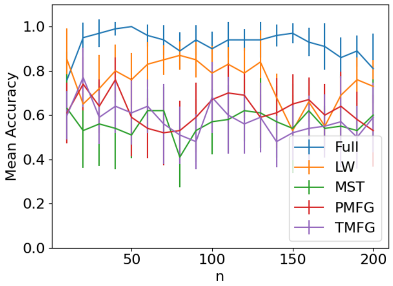

(b) UK $p = 40$

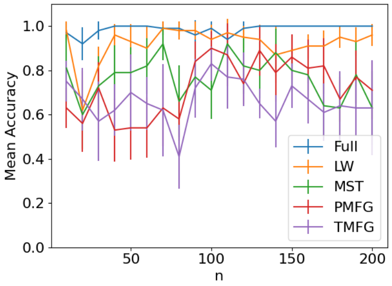

(c) UK $p = 60$

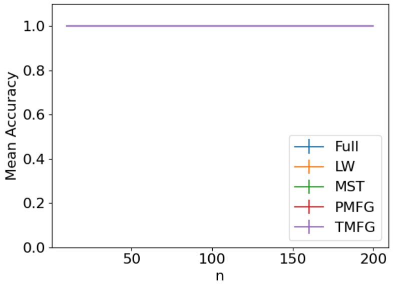

(d) UK $p = 70$

Figure 8: Logistic Regression - Median

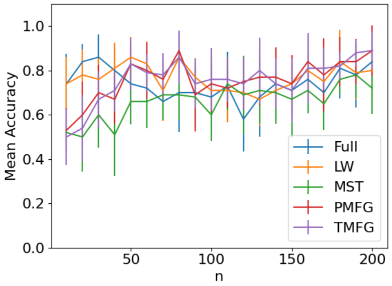

(a) UK $p = 20$

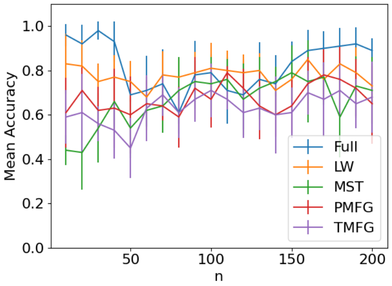

(b) UK $p = 40$

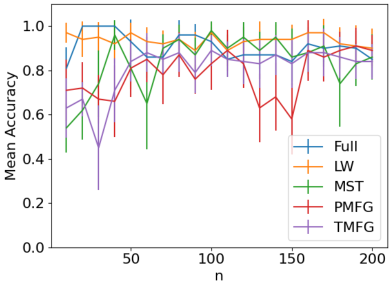

(c) UK $p = 60$

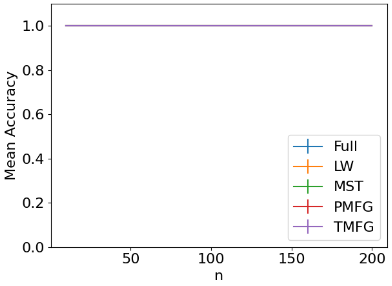

(d) UK $p = 70$

Figure 9: Linear SVM - Max/Min

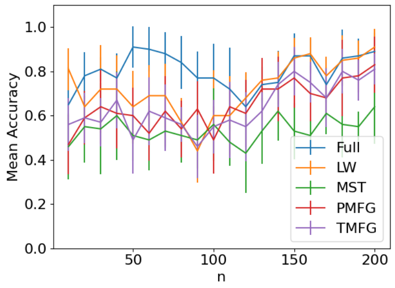

(a) UK $p = 20$

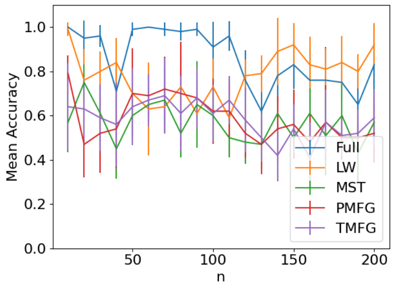

(b) UK $p = 40$

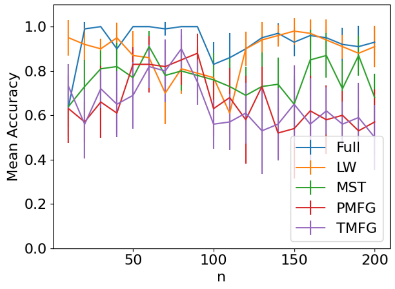

(c) UK $p = 60$

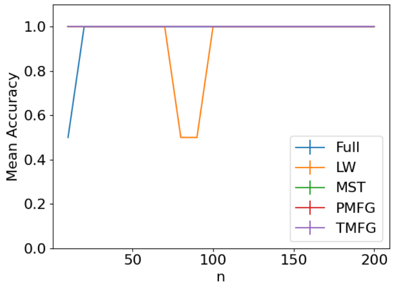

(d) UK $p = 70$

Figure 10: Linear SVM - Median

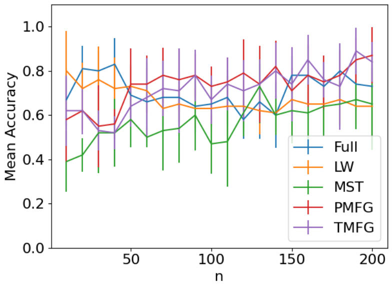

(a) UK $p = 20$

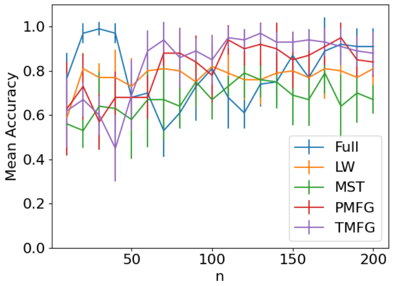

(b) UK $p = 40$

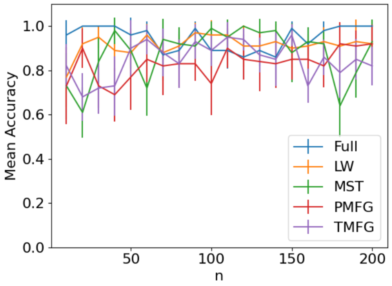

(c) UK $p = 60$

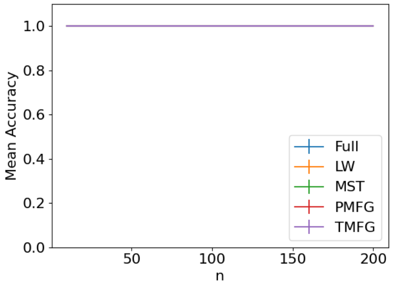

(d) UK $p = 70$

Figure 11: RBF SVM - Max/Min

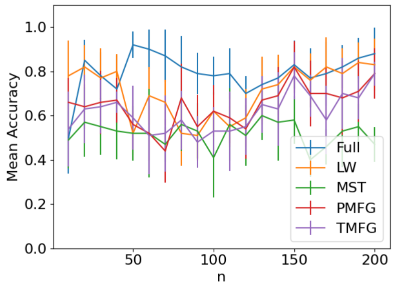

(a) UK $p = 20$

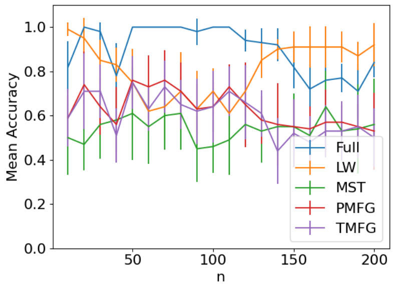

(b) UK $p = 40$

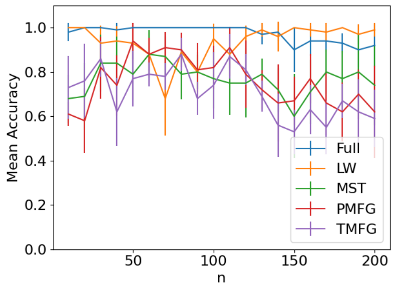

(c) UK $p = 60$

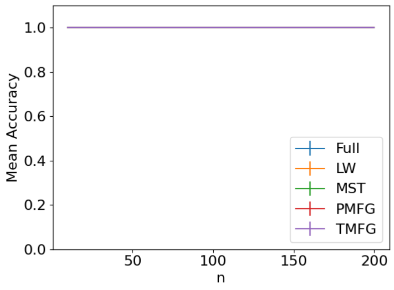

(d) UK $p = 70$

Figure 12: RBF SVM - Median

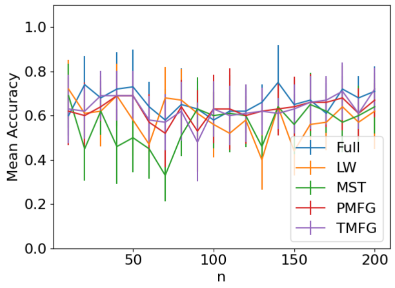

(a) DE $p = 5$

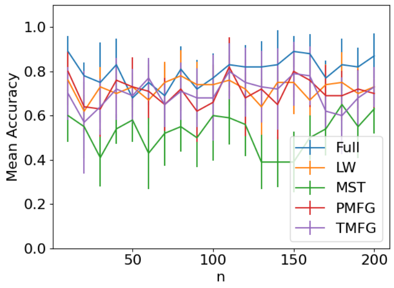

(b) DE $p = 10$

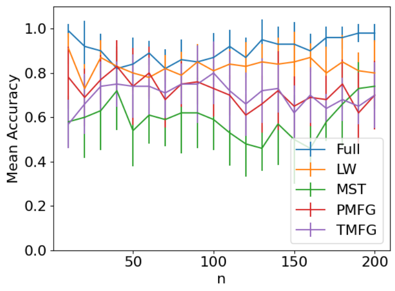

(c) DE $p = 15$

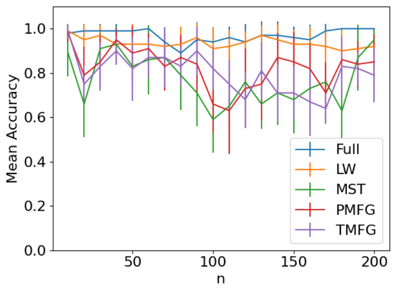

(d) DE $p = 20$

Figure 13: Logistic Regression - Max/Min

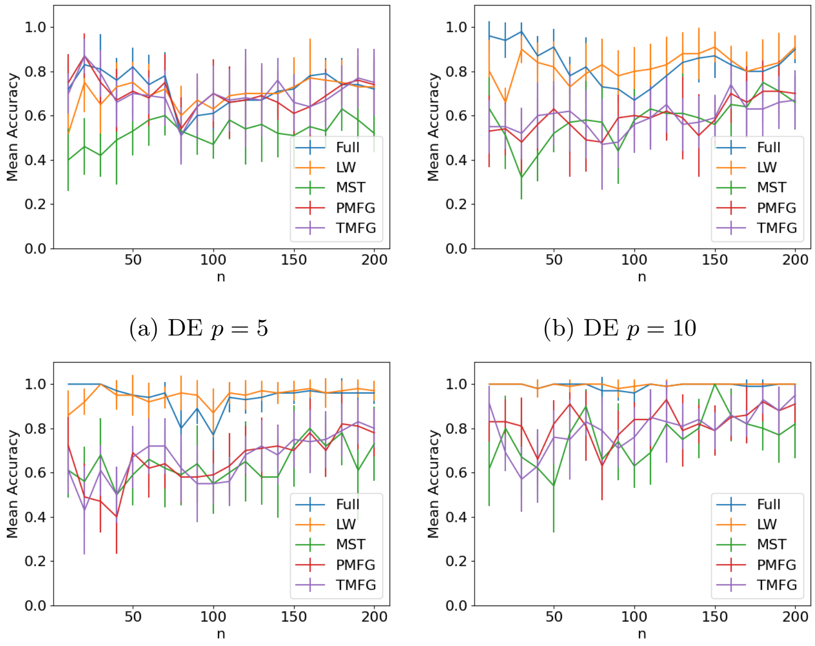

(a) DE $p = 5$

(b) DE $p = 10$

(c) DE $p = 15$

(d) DE $p = 20$

Figure 14: Logistic Regression - Median

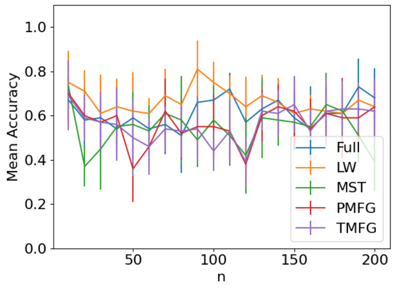

(a) DE $p = 5$

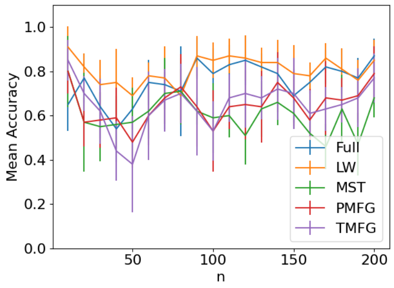

(b) DE $p = 10$

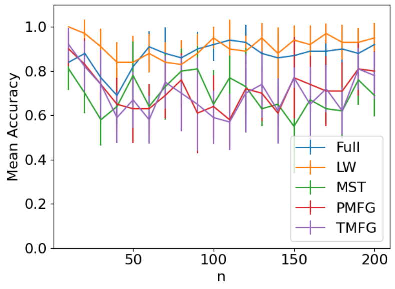

(c) DE $p = 15$

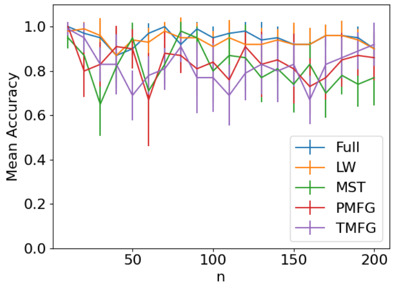

(d) DE $p = 20$

Figure 15: Linear SVM - Max/Min

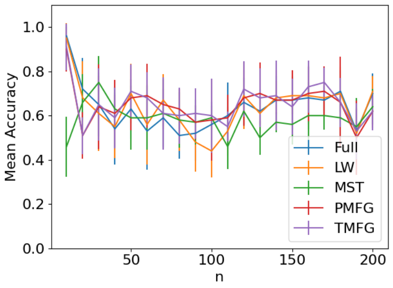

(a) $p = 5$

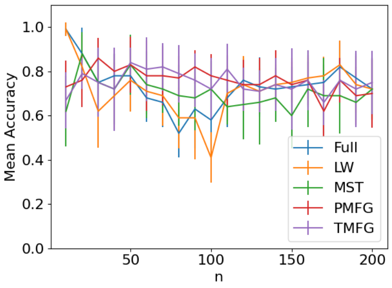

(b) $p = 10$

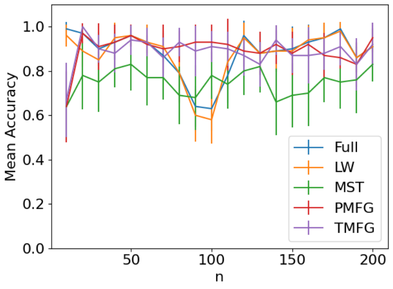

(c) $p = 15$

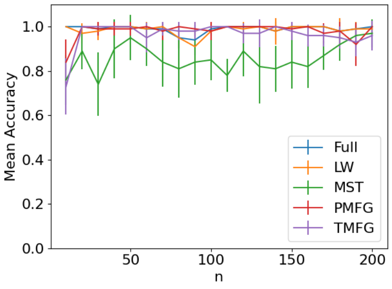

(d) $p = 20$

Figure 16: DE - Linear SVM - Median

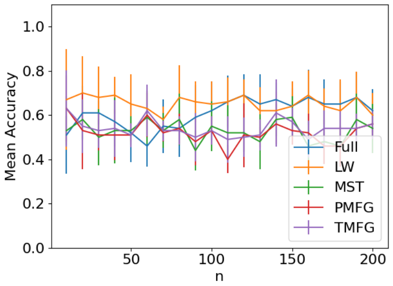

(a) $p = 5$

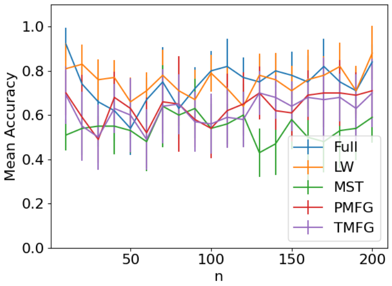

(b) $p = 10$

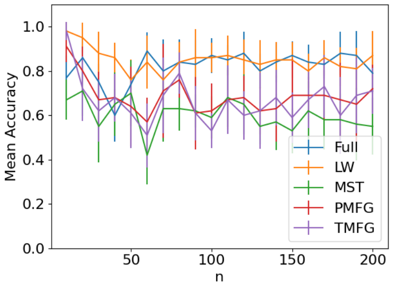

(c) $p = 15$

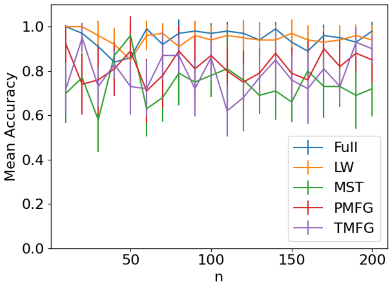

(d) $p = 20$

Figure 17: DE - RBF SVM - Max/Min

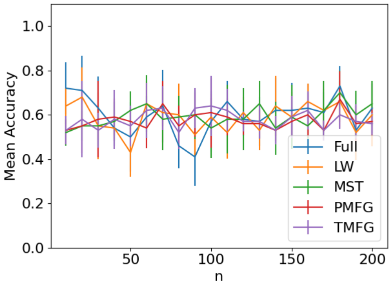

(a) $p = 5$

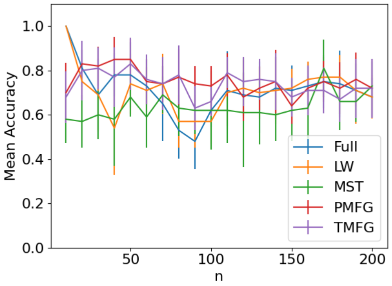

(b) $p = 10$

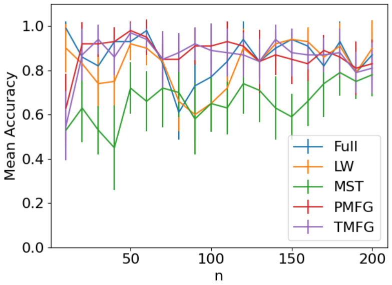

(c) $p = 15$

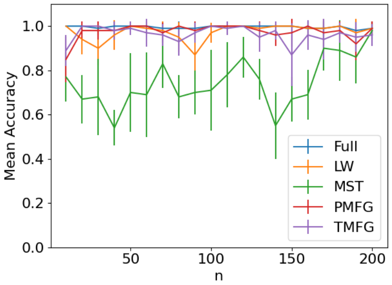

(d) $p = 20$

Figure 18: DE - RBF SVM - Median

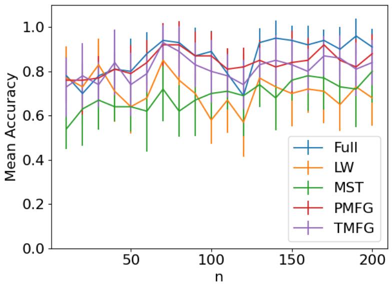

(a) IN $p = 10$

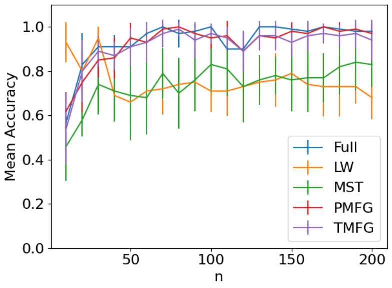

(b) IN $p = 20$

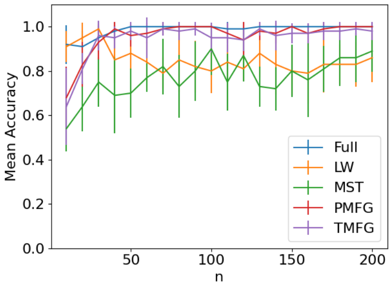

(c) IN $p = 30$

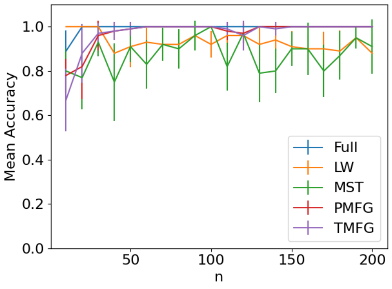

(d) IN $p = 40$

Figure 19: Logistic Regression - Max/Min

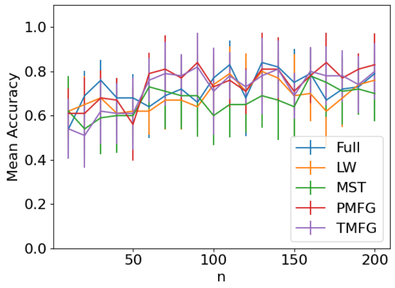

(a) IN $p = 10$

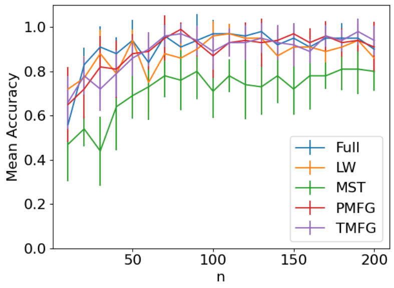

(b) IN $p = 20$

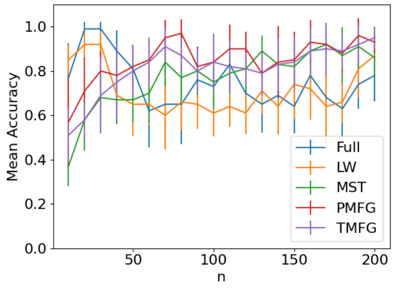

(c) IN $p = 30$

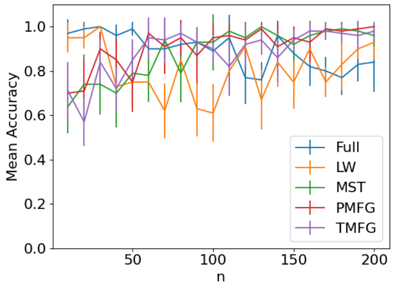

(d) IN $p = 40$

Figure 20: Logistic Regression - Median

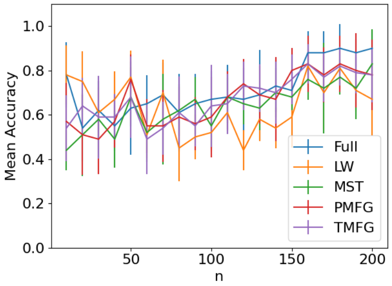

(a) IN $p = 10$

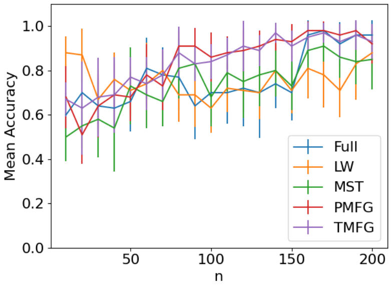

(b) IN $p = 20$

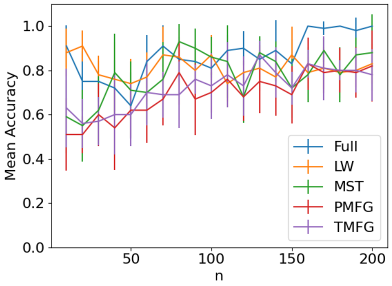

(c) IN $p = 30$

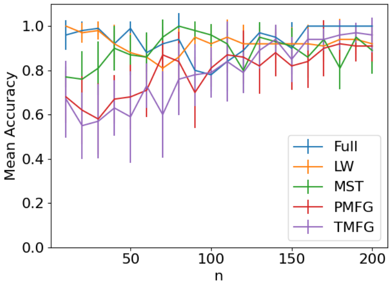

(d) IN $p = 40$

Figure 21: Linear SVM - Max/Min

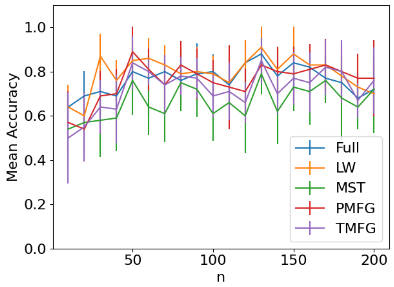

(a) IN $p = 10$

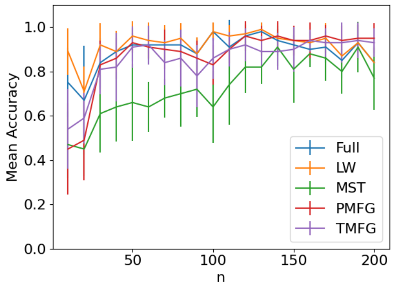

(b) IN $p = 20$

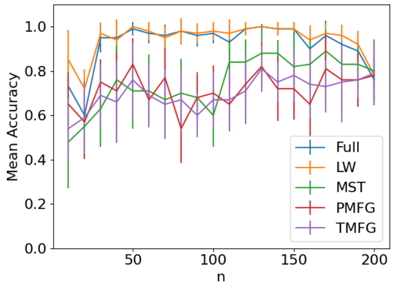

(c) IN $p = 30$

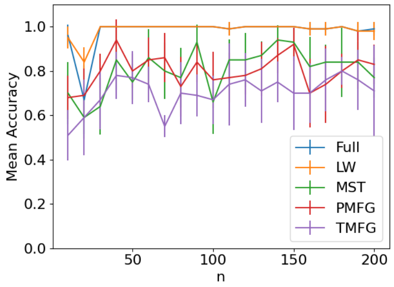

(d) IN $p = 40$

Figure 22: Linear SVM - Median

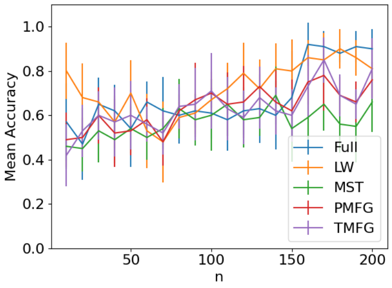

(a) IN $p = 10$

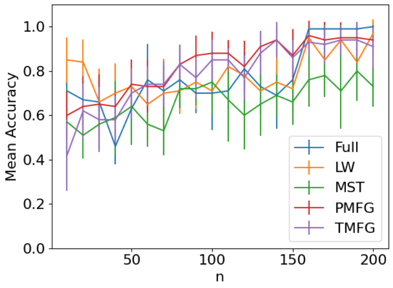

(b) IN $p = 20$

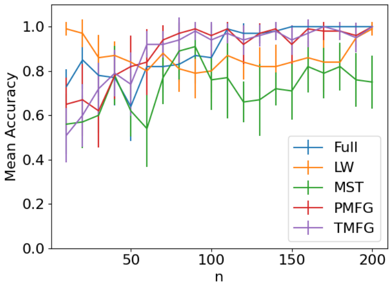

(c) IN $p = 30$

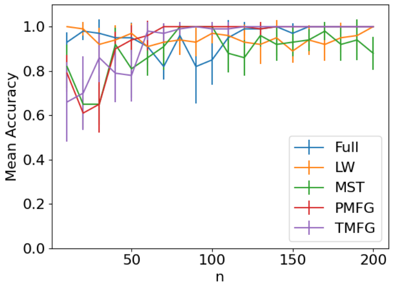

(d) IN $p = 40$

Figure 23: RBF SVM - Max/Min

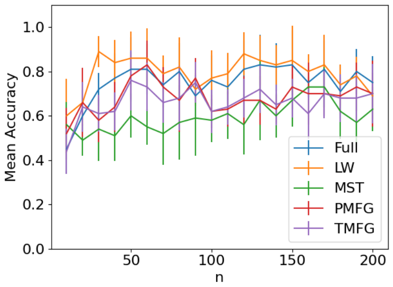

(a) IN $p = 10$

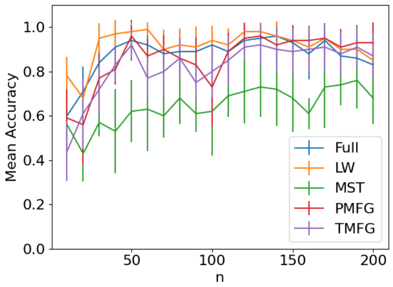

(b) IN $p = 20$

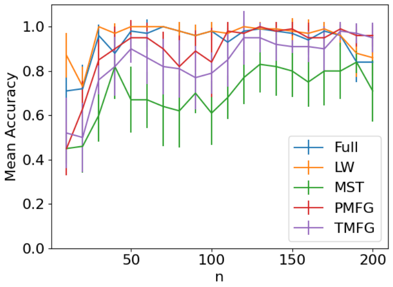

(c) IN $p = 30$

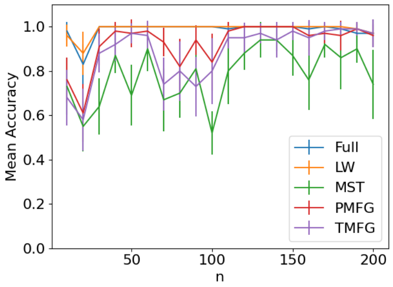

(d) IN $p = 40$

Figure 24: RBF SVM - Median

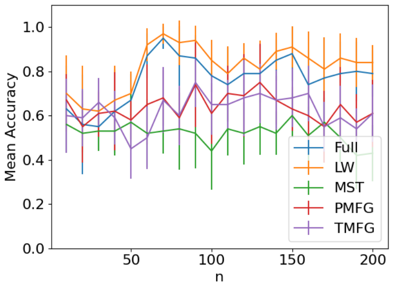

(a) CH $p = 10$

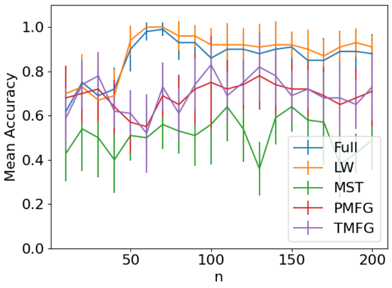

(b) CH $p = 15$

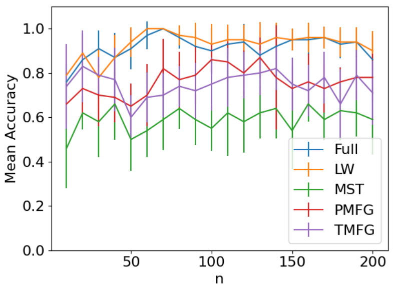

(c) CH $p = 20$

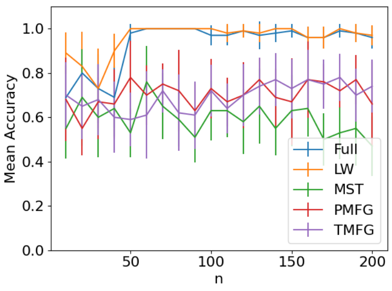

(d) CH $p = 25$

Figure 25: Logistic Regression - Max/Min

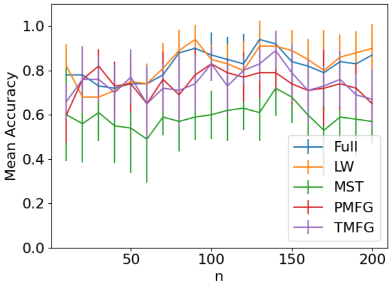

(a) CH $p = 10$

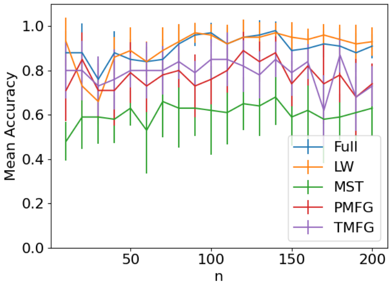

(b) CH $p = 15$

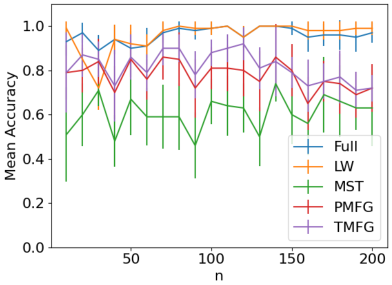

(c) CH $p = 20$

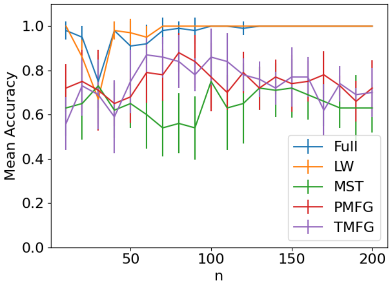

(d) CH $p = 25$

Figure 26: Logistic Regression - Median

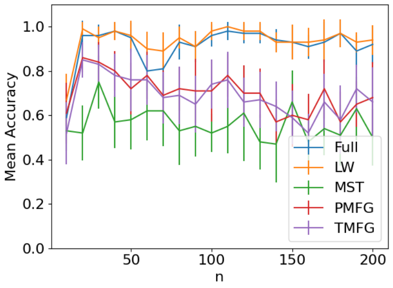

(a) CH $p = 10$

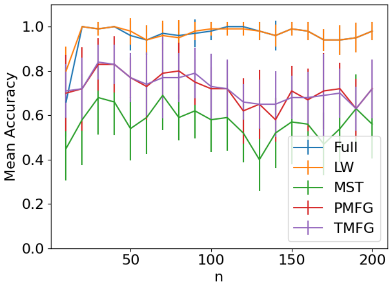

(b) CH $p = 15$

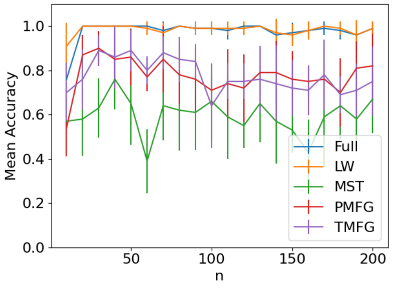

(c) CH $p = 20$

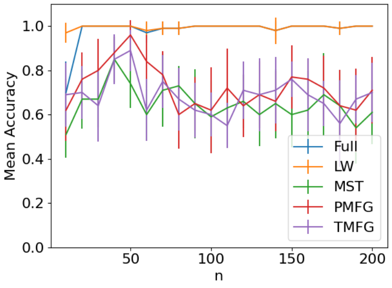

(d) CH $p = 25$

Figure 27: Linear SVM - Max/Min

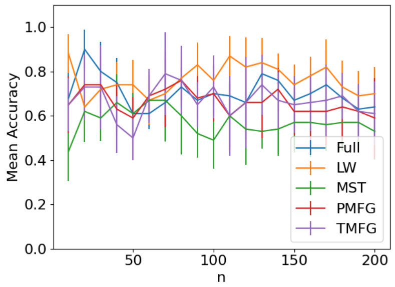

(a) CH $p = 10$

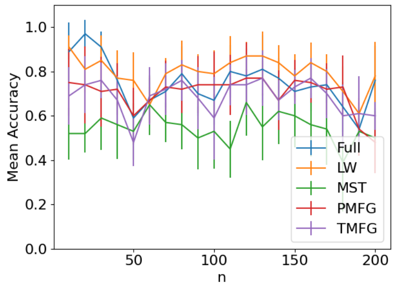

(b) CH $p = 15$

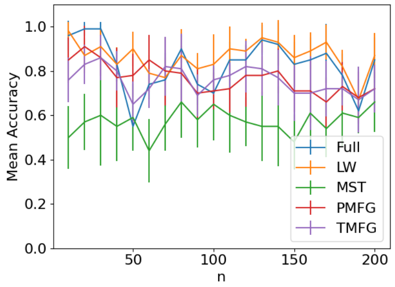

(c) CH $p = 20$

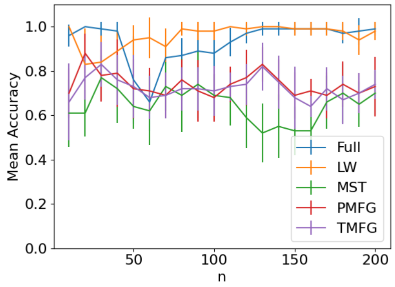

(d) CH $p = 25$

Figure 28: Linear SVM - Median

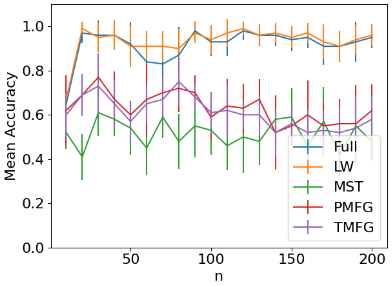

(a) CH $p = 10$

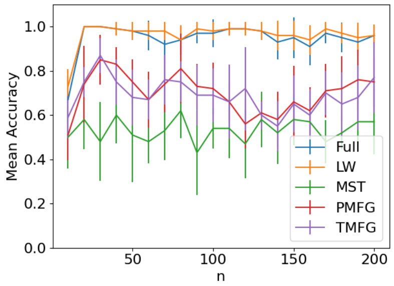

(b) CH $p = 15$

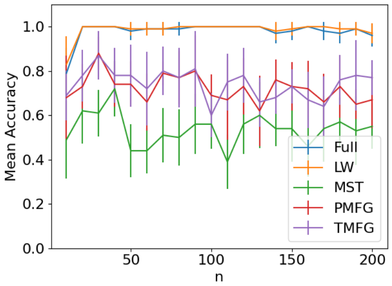

(c) CH $p = 20$

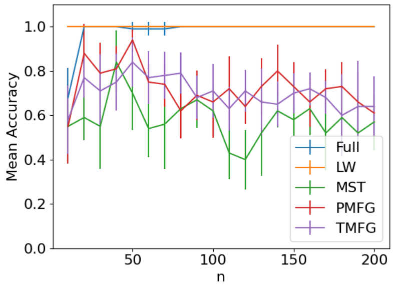

(d) CH $p = 25$

Figure 29: RBF SVM - Max/Min

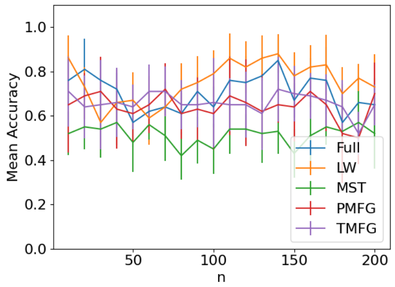

(a) CH $p = 10$

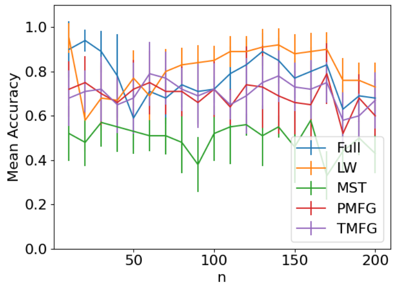

(b) CH $p = 15$

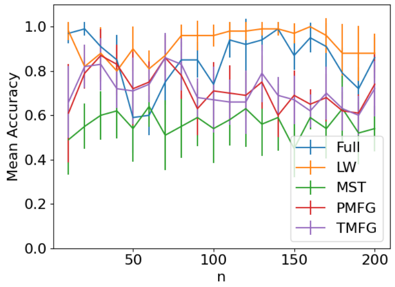

(c) CH $p = 20$

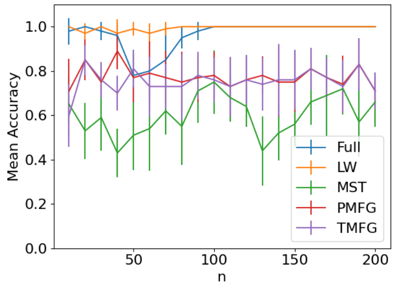

(d) CH $p = 25$

Figure 30: RBF SVM - Median
